# Tanshinone IIA: a Chinese herbal ingredient for the treatment of atherosclerosis

**DOI:** 10.3389/fphar.2023.1321880

**Published:** 2023-12-01

**Authors:** Chunkun Yang, Yanguang Mu, Shuanghong Li, Yang Zhang, Xiaoyuan Liu, Jun Li

**Affiliations:** ^1^ Guang’anmen Hospital, China Academy of Chinese Medical Sciences, Beijing, China; ^2^ Weifang People’s Hospital, Weifang, China; ^3^ Weifang Hospital of Traditional Chinese Medicine, Weifang, China

**Keywords:** tanshinone IIA, vascular endothelial cells, vascular smooth muscle cells, atherosclerosis, plaques, adverse drug reactions, adhesion

## Abstract

Tanshinone IIA (Tan IIA) is a fat-soluble compound extracted from *Salvia miltiorrhiza*, which has a protective effect against atherosclerosis (AS). Tan IIA can inhibit oxidative stress and inflammatory damage of vascular endothelial cells (VECs) and improve endothelial cell dysfunction. Tan IIA also has a good protective effect on vascular smooth muscle cells (VSMCs). It can reduce vascular stenosis by inhibiting the proliferation and migration of vascular smooth muscle cells (VSMCs), and improve the stability of the fibrous cap of atherosclerotic plaque by inhibiting apoptosis and inflammation of VSMCs. In addition, Tan IIA inhibits the inflammatory response of macrophages and the formation of foam cells in atherosclerotic plaques. In summary, Tan IIA improves AS through a complex pathway. We propose to further study the specific molecular targets of Tan IIA using systems biology methods, so as to fundamentally elucidate the mechanism of Tan IIA. It is worth mentioning that there is a lack of high-quality evidence-based medical data on Tan IIA treatment of AS. We recommend that a randomized controlled clinical trial be conducted to evaluate the exact efficacy of Tan IIA in improving AS. Finally, sodium tanshinone IIA sulfonate (STS) can cause adverse drug reactions in some patients, which needs our attention.

## 1 Introduction

Atherosclerotic cardiovascular disease is a major global public health problem, posing a huge threat to people’s health (Libby, 2021). Atherosclerosis (AS) is a chronic, progressive disease that can develop into serious life-threatening complications, including acute myocardial infarction (AMI), ischemic cardiomyopathy and stroke (Gimbrone and García-Cardeña, 2016). Although the medical community has made great progress in the study of the pathogenesis of AS, the morbidity and mortality associated with AS remain high. Medical professionals urgently need drugs with better efficacy to prevent and treat such diseases. Compared with traditional chemical drugs, natural compounds extracted from Chinese herbs have the advantages of fewer side effects and low long-term toxicity. Pharmacological studies have shown that Chinese herbal medicine contains a variety of active ingredients, which play an important role in the prevention and treatment of AS.

**FIGURE.1:**
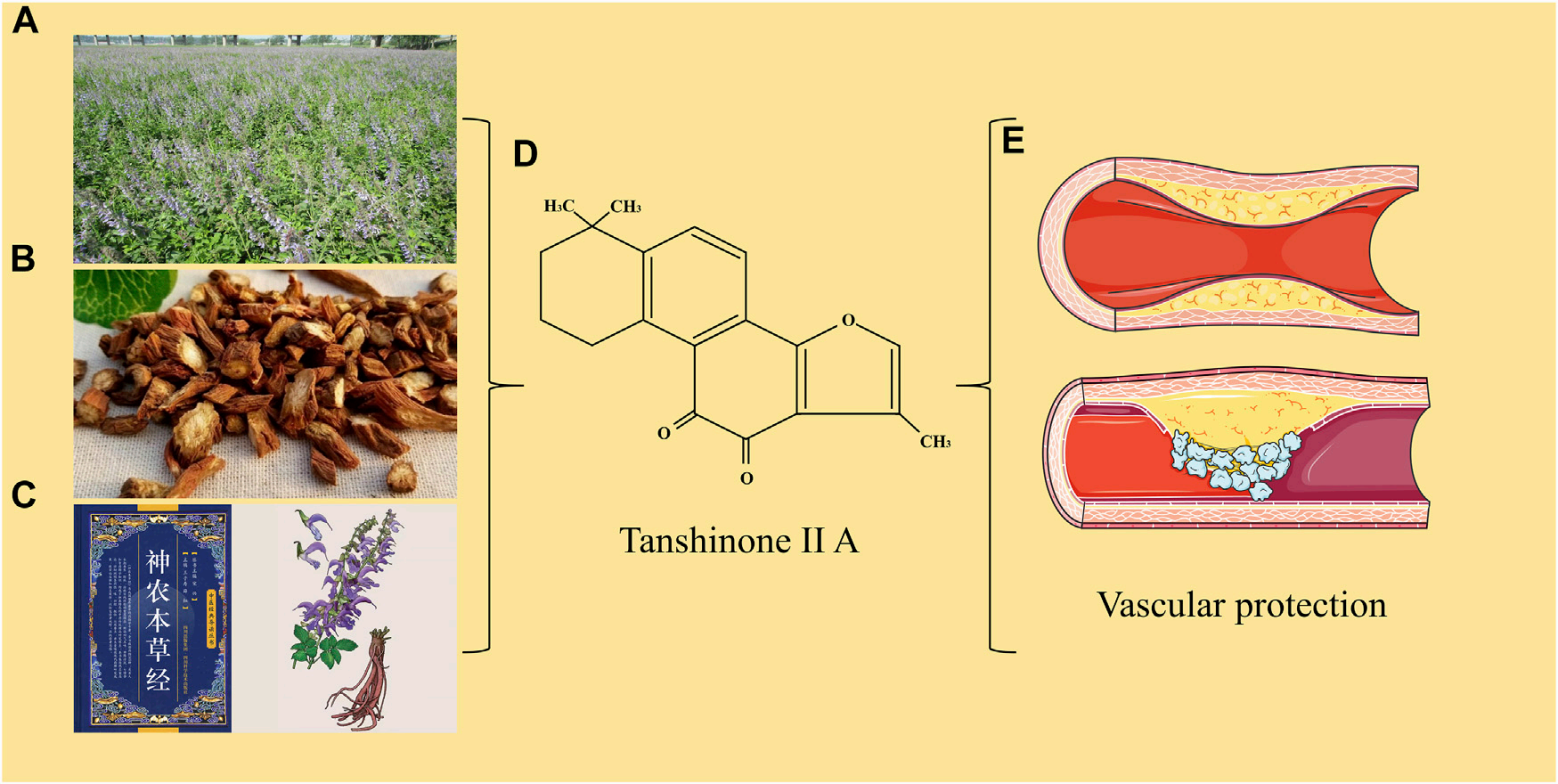
**(A)** Salvia miltiorrhiza. **(B)** Salvia miltiorrhiza. Decoction pieces. **(C)** Shennong’s Classic of Material Medical and picture of Salvia miltiorrhiza. **(D)** Chemical structure of Tanshinone IIA. **(E)** The protection of Tanshinone IIA on blood vessels.

*Salvia miltiorrhiae*, a plant in the labiform family, grows mainly in Hebei, Shanxi, Shaanxi and Shandong provinces of China, and its dried roots are used as Chinese herbs. It is one of the most widely used and oldest traditional Chinese medicine, has the effect of promoting blood circulation and removing blood stasis, and is often used to treat cardiovascular diseases (CVDs). Tanshinone ⅡA (Tan ⅡA) is a fat-soluble active component of *Salvia miltiorrhiza* with a molecular weight of 294.34. It is insoluble or slightly soluble in water and easily soluble in organic solvents such as dimethyl sulfoxide, ethanol, acetone, ether and benzene (Zhang et al., 2019). Previous studies have shown that Tan ⅡA has a variety of beneficial effects on the cardiovascular system, including anti-platelet aggregation, anti-proliferation of vascular smooth muscle cells (VSMCs), coronary artery dilation, and anti-inflammatory effects (Jiao et al., 2013; Ma et al., 2015) (Figure 1). We searched the previous literature and found that the reviews of Tan IIA for the treatment of AS are few and old. In order to better summarize the mechanisms related to the protection of TanⅡA against AS, we searched the relevant studies of TanⅡA again and reviewed them.

## 2 Search strategy

This review was prepared based on recommendations and guidelines from the Preferred Reporting Project for Systematic Review and Meta-Analysis (PRISMA). All relevant articles published in PubMed between the date the database was established and November 2023 were included in this review. We used keywords “Tan IIA”,“tanshinone IIA”,“vascular smooth muscle cells”,“vascular endothelial cells”,“inflammation”,“plaque”,adhesion”, “macrophages”,“lipids”,“atherosclerosis” and “vasodilation ” to search the database. We do an initial screening of the obtained articles by browsing the title and abstract, and then read the full text to incorporate authoritative articles. As a result, we included 69 original documents.

## 3 Tan IIA protects the endothelial cells of blood vessels

Vascular endothelium, the continuous cellular lining of the cardiovascular system, is an important regulatory nodes in this homeostatic network. Ross and Glomset put forward the ‘Response-to-Injury Hypothesis’, which suggests that the early characteristics of AS are the loss of the anatomical integrity of the vascular endothelial (Ross and Glomset, 1976). According to the original statement, The Response-to-Injury Hypothesis assumes that the initial event of AS process was some form of obvious damage to the vascular endothelial cells (VECs), induced by noxious substances (e.g., hyperhomocystemia, oxidized cholesterol, hyperglycemia, constituents of cigarette smoke, etc.) or altered hemodynamic forces. With the expanded awareness of the repertoire of important functions of endothelial cells, the term “endothelial cell dysfunction (ECD)” was introduced into the mainstream of AS research. ECD is associated with nitric oxide (NO) reduction in VECs, Oxidative stress in VEC, proinflammatory activation of VEC, chemokine secretion of VEC, and hemodynamic disturbance (Gimbrone and García-Cardeña, 2016).

**FIGURE 2:**
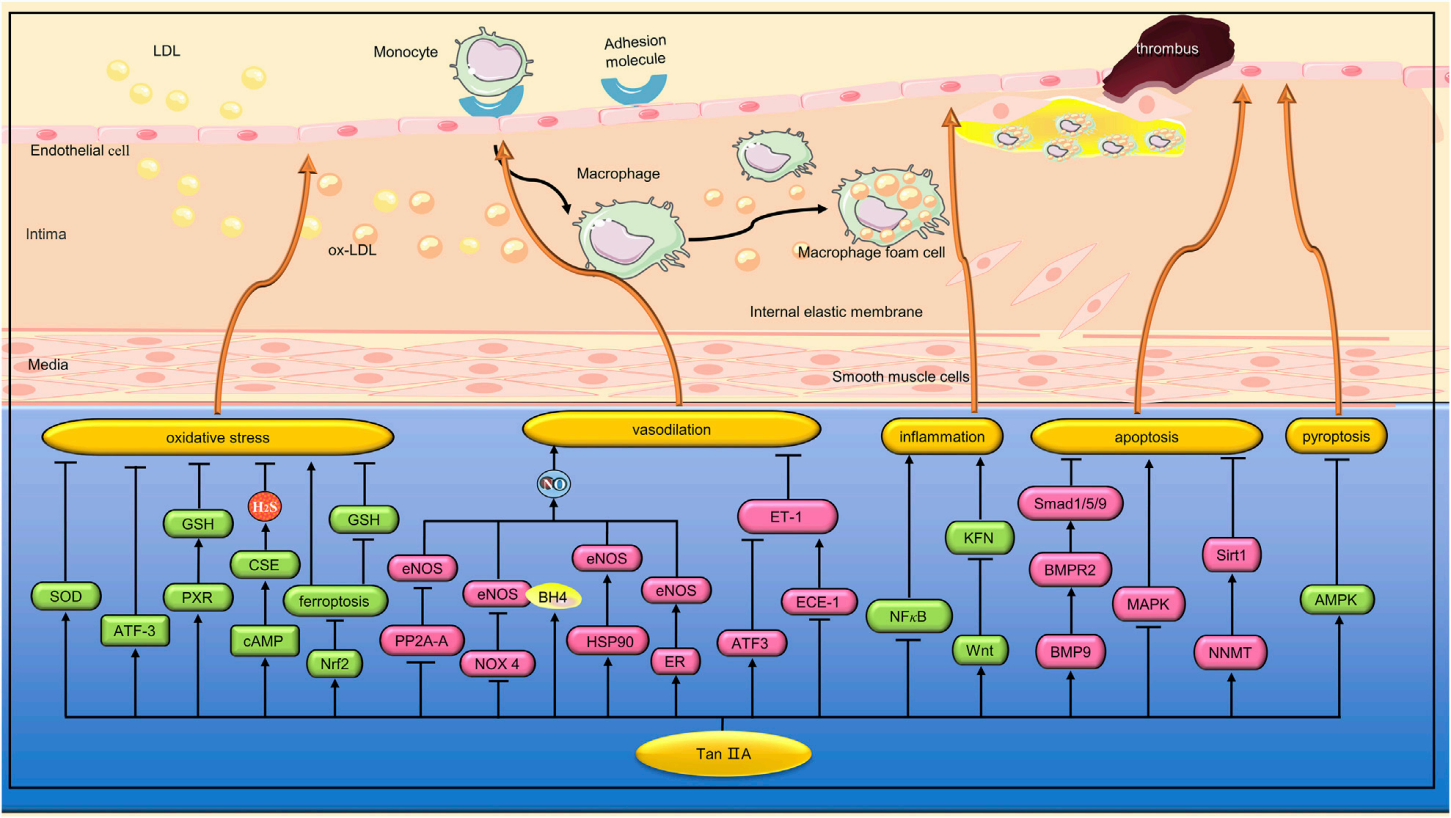
Pharmacological effects of Tan IIA on vascular endothelial cells. Tan IIA protects vascular endothelial cells by anti-inflammation, anti-oxidative stress and anti-apoptosis. In addition, Tan IIA has endothelium-dependent vasodilation and protection of endothelial barrier function.

The damage of VECs is the initial step of AS (Higashi et al., 2009). Oxidative stress is considered to be a key factor in VECs damage. Lin et al. found that Tan IIA (15, 30, and 60 μg/mL) can reduce hydrogen peroxide (H_2_O_2_)-induced human umbilical vein endothelial cell line (ECV-304) cell damage by increasing superoxide dismutase (SOD) activity and inhibiting cluster of differentiation 40(CD40) inflammatory pathway (Lin et al., 2006). In another study, Jia et al. found that Tan IIA (5, 10, and 20 μg/μL) significantly inhibited H_2_O_2_-induced reactive oxygen species(ROS) rise and apoptosis in EA. hy926 cells (Jia et al., 2012). Activated transcription factor (ATF)3 is a stress-induced transcription suppressor (Hashimoto et al., 2002). Chan et al. found that Tan IIA (3 and 10 μM) could inhibit H_2_O_2_-induced apoptosis of human umbilical vein endothelial cells (HUVECs) by up-regulating the expression of ATF3 (Chan et al., 2012). Pregnane X receptor (PXR) is a regulatory factor involved in endogenous and exogenous detoxification. Zhu et al. found that Tan IIA (5,10 and 20 µM) inhibited H_2_O_2_-induced HUVECs apoptosis, inflammation and oxidative stress by activating PXR (Zhu et al., 2017). Hydrogen sulfide (H_2_S), an important gaseous medium, can be synthesized by cystethionine gamma-lyase (CSE). It has a variety of biological functions such as anti-inflammatory, antioxidant, cardiac protection, vasodilation and angiogenesis (Lv et al., 2020). Acrolein, a ubiquitous contaminant in the environment, food and water, can induce oxidative stress through a variety of mechanisms. Yan et al. found that Tan IIA (20 μg/mL) could reduce HUVEC oxidative damage induced by acrolein. The mechanism is related to the upregulation of H_2_S by activation of estrogen receptor (ER)/cyclic adenosine monophosphate (cAMP)/CSE pathway (Yan et al., 2021). Ferroptosis is a form of cell death manifested by the accumulation of iron and ROS (Dixon et al., 2012). Nuclear factor erythroid 2-related factor 2(Nrf2) is a transcription factor that responds to oxidative stress and protects cells from oxidative stress damage. He et al. found that Tan IIA (50 nM) can inhibit ferroptosis by activating Nrf2, inhibit the increase of lipid peroxidation and the decrease of GSH level, thus alleviating the death of human coronary artery endothelial cells (HCAECs) caused by ferroptosis inducers (He et al., 2021). (Figure 2)

ECD is an important feature of many CVDs (Thum et al., 2007). Endothelial nitric oxide synthase (eNOS) is one of the key enzymes in maintaining vascular endothelial homeostasis through the production of NO. Li et al. found that Tan IIA (0.5 mg/kg) inhibited the decrease of eNOS expression and NO production in the aorta of diabetic rats, thereby improving the impaired endothalium-dependent vasodilation function (Li et al., 2015). Previous studies have shown that regulation of eNOS expression can occur at the transcriptional, post-transcriptional, and post-translational levels (Stangl et al., 2004). Li et al. found that the regulation of Tan IIA on eNOS expression mainly occurred at the post-translation level. Protein phosphatase 2A (PP2A) is a serine/threonine phosphatase that can induce dephosphorylation of eNOS. Li et al. found that Tan IIA did not affect the expression and activity of PP2A, but significantly inhibited the translocation of PP2A-A from cytoplasm to membrane and impaired PP2A-A/eNOS interaction, thereby preventing eNOS dephosphorylation (Li et al., 2015). Uncoupling of eNOS can occur under some pathological conditions, resulting in the production of superoxide instead of NO (Förstermann, 2010). Tetrahydrobiopterin (BH4), a key cofactor in eNOS activity, inhibits eNOS uncoupling, while dihydrobiopterin (BH2), an oxidized form of BH4, can cause eNOS dysfunction (Noguchi et al., 2011). Zhou et al. found that Tan IIA (10 μM) inhibited eNOS uncoupling in EA. hy926 cells induced by high glucose (35 mM). The mechanism is related to the inhibition of nicotinamide-adenine dinucleotide phosphate (NADPH) oxidase 4 (NOX4) activity and expression, and the increase of the ratio of BH4 to BH2. Heat shock protein 90(HSP90) is an important partner in regulating eNOS function. Zhou et al. found that Tan IIA can also inhibit eNOS uncoupling by promoting the expression of HSP90 (Zhou et al., 2012). The interaction between estrogen and ER can lead to transcriptional activation of eNOS. Previous studies have shown that Tan IIA, as a phytoestrogens, can activate ER (Fan et al., 2009). Fan et al. found that Tan IIA (10 μM) mitigated the constriction of the aortic ring induced by phenylephrine in rats. *In vitro* studies, Tan IIA can increase NO release from cardiac microvascular endothelial cells (CMECs) by activating ER, promoting eNOS gene expression, extracellular signal-regulated kinase 1/2 (ERK1/2) phosphorylation, and Ca^2+^ mobilization (Fan et al., 2011). Endothelin-1 (ET-1), a peptide composed of 21 amino acids, is one of the most potent vasoconstricting peptides in humans. ATF3 is a stress-induced transcription suppressor. Hong et al. found that Tan IIA (3 and 10 μmol⁄L) inhibited strain-induced ET-1 expression by increasing NO and upregating ATF3 in HUVEC (Hong et al., 2012). ET-1 has a high affinity with endothelin-A receptor (ET_A_) and can mediate vascular constriction (Yakubu and Leffler, 2002). However, ET-1 has a low affinity with endothelin-B receptor (ET_B_) and can mediate vasodilation (Clozel et al., 1992). Large ET-1 is a precursor of endothelin and does not bind to any known ET receptors. When large ET-1 is cut by endothelin-converting enzyme-1 (ECE-1), ET-1 with vasoconstrictive activity is formed. Tang et al. found that Tan IIA (10 and 20 μg/mL) can reduce the expression of ET-1 in tumor necrosis factor-α(TNF-α)-induced brain microvascular endothelial cells (BMVECs) by inhibiting ECE-1 synthesis. In addition, Tan IIA also inhibited the expression of ET_A_ mRNA and increased the expression of ET_B_ mRNA in TNF-α-induced BMVEC (Tang et al., 2007). Obstructive sleep apnea-hypopnea syndrome (OSAHS) is closely associated with the development of ECD. Chen et al. simulated OSAHS-related episodic hypoxemia by subjecting SD rats to chronic intermittent hypoxia (CIH). Tan IIA (20 mg/kg) was found to improve ECD in CIH rats by inhibiting ET-1 expression, decreasing ET_A_ receptors, increasing ET_B_ receptors, and increasing NO formation (Chen et al., 2017). In another study, Wang et al. found that STS (12.5 µM) can inhibit hypoxia-induced apoptosis of pulmonary microvascular endothelial cells (PMVECs) by regulating the bone morphogenetic protein 9 (BMP9)/bone morphogenetic protein receptor type 2 (BMPR2)/mothers against decapentaplegic homolog 1/5/9(Smad1/5/9) signaling pathway (Wang et al., 2022). (Figure 2)

VECs play an important role in immune defense and inflammatory response. Lipopolysaccharide (LPS) activates the innate immune response, leading to the production of a large number of pro-inflammatory cytokines, which in turn promote vascular permeability. In a study, Cheng et al. found that STS (20 and 40 mg/mL) can inhibit LPS-induced TNF-α and interleukin (IL)-1β expression by inhibiting nuclear factor kappa-B(NF-κB) activation, thereby reducing the inflammatory response of HUVEC (Cheng et al., 2018). Studies have shown that endothelial progenitor cells (EPCs) play a key role in endothelial repair by differentiating into mature VECs and releasing protective paracrine factors (Yang et al., 2013). Wang et al. found that Tan IIA (10 and 20 µM) reversed TNF-α-induced damage to EPCs proliferation, migration, adhesion, and angiogenesis. In addition, Tan IIA inhibits TNF-α-induced EPCs secretion of inflammatory cytokines (Wang et al., 2015). (Figure 2)

The development of diabetes mellitus (DM) is a chronic inflammatory process. Fractalkine, a chemokine, is strongly associated with diabetic nephropathy and coronary heart disease (Bergmann and Sypniewska, 2014). In addition, the Wnt signaling pathway plays a considerable role in the metabolic dysfunction caused by DM (Hisa et al., 2008). Li et al. found that STS (5 and 20 mg/L) can inhibit the expression of fractalkine by activating the classical Wnt pathway, thereby reducing the inflammation of HUVEC cells induced by high glucose (33.3 mmol/L) (Li et al., 2015). Methylglyoxal (MGO),a major precursor of advanced glycation end products (AGEs), plays an important role in the progression of vascular complications in diabetes (Ogawa et al., 2010). Zhou et al. found that Tan IIA (10,20 and 30 μg/mL) alleviated MGO-induced human brain microvascular endothelial cells (HBMEC) apoptosis and oxidative stress, and the mechanism was related to inhibiting mitogen-activated protein kinase (MAPK) activation (Zhou et al., 2015). Hyperhomocysteinemia (HHcy) has been shown to be an independent risk factor for CVDs. Zhou et al. found that STS (25, 50 and 100 µM) significantly reversed homocysteine (Hcy)-induced HUVECs damage. The mechanism is related to the activation of nicotinamide N-methyltransferase (NNMT)/sirtuin1(Sirt1) signaling pathway (Zhou et al., 2023). Pyroptosis is a newly discovered form of programmed cell death driven by the NLR family pyrin domain containing 3(NLRP3) inflammasome. Zhu et al. found that STS (20 mg/kg) inhibited the upregulation of typical pyroptosis signals in the aortic wall of ApoE^−/−^ mice, thereby enhancing plaque stability. *In vitro* experiments, STS (10, 20, and 40 μM) inhibited HUVECs pyroptosis induced by cholesterol crystals (CC) by a mechanism related to activation of the Adenosine 5‘-monophosphate (AMP)-activated protein kinase (AMPK) signaling pathway (Zhu et al., 2022). (Figure 2)

## 4 Regulation of vascular smooth muscle cells by Tan IIA

VSMCs turnover is low in normal blood vessel walls, but increased proliferation of VSMCs is observed in early AS or when blood vessels are damaged (Lutgens et al., 1999). Previous studies have shown that abnormal proliferation of VSMC is closely associated with some CVDs, such as and intrastent restenosis (Inoue and Node, 2009). Reducing the abnormal proliferation of VSMC is a therapeutic strategy to inhibit intravascular stenosis. Apoptosis of VSMCs and destruction of the extracellular matrix (ECM) can lead to thinning of the fiber cap and increase the risk of plaque rupture, which can induce AMI or stroke (Bennett et al., 2016). VSMC apoptosis in AS can also release multiple types of ILs, which can induce inflammation (Clarke et al., 2010).

**FIGURE 3:**
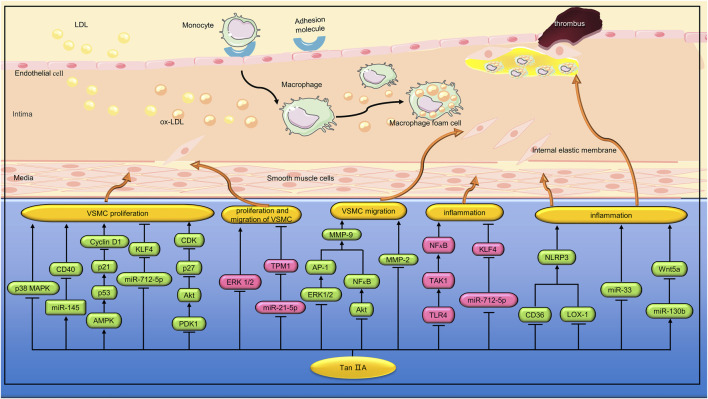
Pharmacological effects of Tan IIA on vascular smooth muscle cells and macrophages. Tan IIA inhibits proliferation, migration and inflammation of vascular smooth muscle cells. Tan IIA also inhibits macrophage inflammation.

Percutaneous coronary intervention (PCI) is an effective treatment for coronary artery stenosis, but intrastent restenosis limits long-term success. Li et al. found that Tan IIA (40 and 120 mg/kg) can inhibit intimal thickening of the carotid artery with balloon injury in rats. *In vitro* studies, Tan IIA (0.1, 0.25, 0.5, and 1.0 g/mL) inhibited VSMC proliferation and cell cycle progression induced by fetal bovine serum (FBS) through a mechanism related to inhibition of ERK1/2 phosphorylation (Li et al., 2010). Diabetes can increase the risk of cardiovascular complications, such as (Berezin et al., 2016). AGEs formed by persistent hyperglycemia play an important role in the pathogenesis of chronic complications of diabetes (Vlassara et al., 1994). Lu et al. found that Tan IIA (10 μmol/L) inhibited AGEs-induced proliferation and migration of VSMCs by inhibiting the ERK1/2 signaling pathway (Lu et al., 2018). Angiotensin II(Ang II) is the main effector hormone of renin-angiotensin-aldosterone system (RAAS). Lu et al. found that Tan IIA (5 and 10 μg/mL) alleviated Ang II-induced VSMCs proliferation and autophagy by inhibiting the phosphorylation of p38 MAPK (Lu et al., 2019). (Figure 3)

AMPK is a physiological sensor of cellular energy status and plays a key role in regulating glucose metabolism (Misra, 2008). Previous studies have shown that high glucose can induce the proliferation and migration of VSMC (Zhu et al., 2009). Wu et al. found that STS (10 µM) inhibited the proliferation of VSMC induced by high glucose (25 mM) by activating AMPK and up-regulating the expression of cell cycle regulatory proteins p53 and p21. In addition, STS can reduce VSMC migration by inhibiting the expression and activity of matrix metalloproteinase 2 (MMP2) (Wu et al., 2014). MicroRNA (miRNA) is a class of non-coding RNA molecules with a length of about 22 nucleotides. Tropomyosin 1 (TPM1), a member of the actin-binding protein family, plays a key role in the regulation of skeletal and cardiac muscle contraction. Previous studies have shown that TPM1 is an effective target of miR-21-5p in vascular cells (Wang et al., 2011). Jia et al. found that Tan IIA (10 µM) can reduce hyperglucose (25 mM)-induced human aortic smooth muscle cell (HASMC) proliferation and migration by inhibiting the miR-21-5p/TPM1 signaling pathway (Jia et al., 2019). Hcy can promote the progression of AS by inducing excessive proliferation and phenotypic conversion of VSMCs. CD40 is involved in regulating the proliferation, migration and apoptosis of VSMCs (Li et al., 2016). Li et al. found that Tan IIA (3 and 10 μmol/L) inhibited hcy (200 μmol/L) induced VSMCs proliferation through the miR-145/CD40 signaling pathway (Li et al., 2020). Transient receptor potential cation channel subfamily C member 3 (TRPC3) is an ion channel protein that has the function of regulating the cardiovascular system. Li et al. found that Tan IIA (10 μg/mL) can inhibit ox-LDL-induced VSMC proliferation and migration by regulating the miR-137/TRPC3 signaling pathway (Li et al., 2023). Hyperplasia of basilar vascular smooth muscle cells (BASMCs) caused by high blood pressure can lead to vascular remodeling, which increases the risk of stroke. Yu et al. found that Tan IIA (5 μmol/L) can prevent ET-1 induced BASMCs proliferation by inhibiting 3’-phosphoinostitide dependent kinase (PDK1)/Akt pathway and regulating the expression of cyclin D1 and p27 (Yu et al., 2015). MMPs can contribute to VSMC migration by degrading the surrounding ECM (Newby and Zaltsman, 2000). In the promoter region of MMP-9 gene, there is one NF-κB binding site and two activator protein-1 (AP-1) binding sites. Jin et al. found that Tan IIA (100 µM) can reduce MMP-9 activity by inhibiting ERK1/2/AP-1 and Akt/NF-κB signaling pathways, thereby inhibiting HASMC migration (Jin et al., 2008). (Figure 3)

VSMCs can promote the progression of AS by up-regulating inflammatory cytokines. LPS is an endotoxin that increases the expression of various inflammatory factors in VSMC. Toll-like receptors 4 (TLR4), a member of the TLR family, binds to LPS to induce innate and adaptive immune responses (Minguet et al., 2008). Transforming growth factor-β(TGF-β)-activated kinase 1 (TAK1) is a downstream molecule of TLR4 signaling pathway, which can cause the activation of NF-κB and promote the expression of various inflammatory factors. Meng et al. found that TanⅡA (25, 50, and 100 μmol/L) can reduce LPS (1 μg/mL)-induced inflammatory response in VSMC by inhibiting the TLR4/TAK1/NF-κB signaling pathway (Meng et al., 2019). Krüppel-like factor 4(KLF4) can regulate phenotypic transformation and regulates inflammatory response and proliferation of VSMC during cardiovascular remodeling (Hartmann et al., 2016). Qin et al. found that Tan ⅡA (1.0 μg/mL) could inhibit TNF-α-induced inflammation and proliferation of VSMC, and its mechanism is related to inhibiting the expression of miR-712-5p, thereby up-regulating KLF4 and down-regulating IL-1β, IL-6 and TNF-α. *In vivo* studies, TanⅡA (10 mg/kg/d) can reduce vascular inflammation and intimal hyperplasia induced by carotid artery ligation in C57BL/6 mice by inhibiting the expression of miR-712-5p (Qin et al., 2020). The main function of mature VSMC is systolic regulation. However, VSMC can undergo phenotypic transitions from the differentiation stage to the dedifferentiation stage, thus promoting the proliferation and migration of VSMC (Alexander and Owens, 2012). Lou et al. found that TanⅡA (1 μM) can regulate the phenotypic transformation of VSMC induced by platelet-derived growth factor (PDGF) and inhibit its proliferation, and the mechanism is related to the enhancement of KLF4 expression (Lou et al., 2020). (Figure 3)

VSMC, as a component of plaque cap, is related to plaque stability. Excessive apoptosis of VSMC can lead to atherosclerotic plaque rupture (Clarke et al., 2006). Wang et al. found that Tan ⅡA (40 and 80 μM) inhibited oxidized low-density lipoprotein (ox-LDL, 50 μg/mL)-induced apoptosis of VSMCs. *In vivo* studies, Tan IIA (30 mg/kg) also inhibited VSMCs apoptosis in the aortic root of ApoE^−/−^ mice (Wang et al., 2017). Abdominal aortic aneurysm (AAA) is a cardiovascular disease with a mortality rate of up to 90%. Previous studies have shown that AAA is related to inflammation, VSMC loss, elastin and collagen destruction (Longo et al., 2002). Shang et al. found that Tan IIA (2 mg/rat/d) can inhibit the aortic size of SD rats induced by elastase. The mechanism may be related to reducing the overexpression of MMP-2, MMP-9, MCP-1 and inducible nitric oxide synthase (iNOS), and inhibiting the depletion of elastic fibers and VSMC (Shang et al., 2012). (Figure 3)

## 5 Tan IIA reduces atherosclerotic inflammation

In the past, the medical community believed that AS was a simple disease caused by the proliferation of VSMC and lipid accumulation in the artery wall. Now, it is generally believed that immunity and inflammation play an important role in the pathological process of AS (Major and Harrison, 2011). In the early stage of AS, the causes of AS are related to VECs injury, abnormal lipid metabolism and hemodynamic injury. When VECs are activated, they express inflammatory factors such as monocyte chemotactic protein-1 (MCP-1), IL-8, vascular cell adhesion molecule-1(VCAM-1), intracellular adhesion molecule-1(ICAM-1), platelet selectin (P-selectin), endothelial selectin (e-selectin), which attract monocytes to bind to VECs, and inflammation begins (Chistiakov et al., 2018). In the middle stage of AS, abnormal activation of macrophages in atherosclerotic plaques and uncontrolled production of pro-inflammatory factors mediate the progression of AS. In the late stage of AS, a large number of macrophages infiltrate the blood vessel wall, degrade ECM in plaque, and cause plaque rupture (Nagy et al., 1998).

The inflammasome is a group of polymeric protein complexes involved in the processing and maturation of inflammatory cytokines IL-1β and IL-18 (Latz et al., 2013). NLRP3 is one of the most characteristic inflammatory bodies. Wen et al. found that Tan IIA (10 μg/mL) can reduce the activation of NLRP3 induced by ox-LDL by inhibiting the expression of lectin-like ox-LDL receptor-1 (LOX-1) and cluster of differentiation 36(CD36) in bone marrow-derived macrophages (BMDMs). *In vivo* studies, Tan IIA (20 mg/kg/day) inhibited the production of IL-1β and IL-18 in aortic tissue and serum of ApoE^−/−^ mice fed a high cholesterol diet (HCD) (Wen et al., 2020). MiRNA is a small non-coding RNA, approximately 22 nucleotides in length, that is involved in regulating ox-LDL-mediated signaling in macrophages (Zhang and Wu, 2013). Yang et al. found that Tan IIA (10 μM) inhibited ox-LDL-induced proinflammatory cytokine (IL-1β, IL-6, and TNF-α) secretion by down-regulating miR-33 in THP-1 macrophages (Yang et al., 2019). Previous studies have shown that Wnt5a can participate in the progression of AS by regulating inflammatory response (Bhatt and Malgor, 2014). Inhibition of Wnt5a can reduce foam cell formation and lipid accumulation in atherosclerotic plaques (Ackers et al., 2018). Yuan et al. found that Tan IIA (5 and 10 μM) inhibited lipid deposition and inflammation in ox-LDL-treated THP-1 macrophages by regulating the miR-130b/Wnt5a pathway (Yuan et al., 2020). (Figure 3)

**FIGURE 4:**
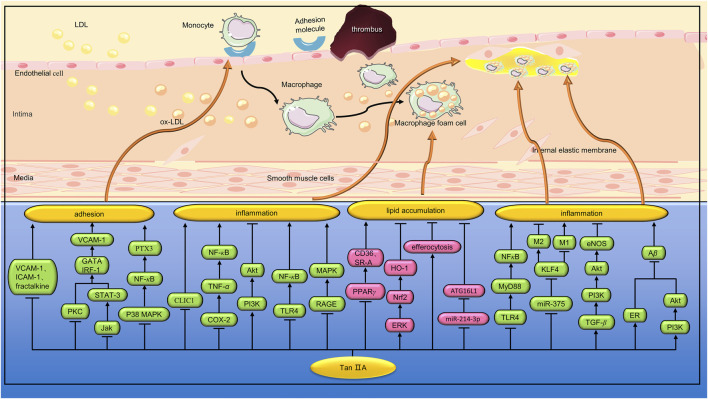
Pharmacological effects of Tan IIA on macrophage inflammation, adhesion and lipid deposition. Tan IIA can reduce macrophage inflammation, inhibit lipid deposition, and inhibit the adhesion of macrophages to vascular endothelial cells.

The inflammatory response of AS is mediated by the interaction between blood vessel wall cells and white blood cells (Campbell and Campbell, 1986). Li et al. established an IL-1β-induced VEC-VSMC-monocyte co-culture model to study the pathogenesis of AS. It was found that Tan IIA (10 μM) inhibited the amount of monocyte adhered to the VEC surface and decreased the expression of TNF-α and MCP-1 secreted by VEC, as well as TGF-β1 and MMP-2 in VSMC supernatant (Li Y. et al., 2015). Amyloid β peptide (Aβ) is composed of 36–43 amino acids and is the proteolytic product of amyloid precursor protein (APP). Previous studies have shown that blood Aβ is closely related to the inflammatory pathology of AS (Kokjohn et al., 2011). Shi et al. found that Tan IIA (5 µM) inhibited the production of Aβ in platelets, and the mechanism was related to the activation of ER and the upregulation of PI3K/Akt pathway (Shi et al., 2014). Estrogen plays a protective role in AS. Tan IIA has an active conformation similar to 17 beta-estradiol, binding to ERs with high affinity. Liu et al. found that Tan IIA (30 and 60 mg/kg/d) can exert anti-AS inflammatory effects in ApoE^−/−^ ovariectomized mice by inhibiting p-ERK1/2 expression and activating ERs (Liu et al., 2015). Circular RNA (CircRNA) is a kind of long non-coding RNA (lncRNA), which can promote messenger RNA (mRNA) transcription by binding with miRNA (Jin et al., 2016). Lan et al. found that STS (400 and 800 μM) inhibited LPS-induced inflammatory responses in RAW264.7 cells by a mechanism related to upregulation of circ-Sirt1 and subsequent blocking of NF-κB from entering the nucleus (Lan et al., 2022). (Figure 4)

Previous studies have shown that rupture of atherosclerotic plaques is a major cause of death in cardiovascular disease. Inflammation can lead to plaque progression and increased vulnerability. MMP-2 and MMP-9 are the major proteases that degrade collagen during atherosclerotic plaque instability (Pasterkamp et al., 2000). Xu et al. found that Tan IIA (10 and 30 mg/kg/d) reduced the size of aortic sinus AS lesions in HCD-fed apoE^−/−^ mice. In addition, Tan IIA can also increase plaque stability by reducing necrotic nuclei and macrophage infiltration, and increasing VSMC and collagen content. *In vitro* studies have shown that Tan IIA (1 and 10 μM) can inhibit the expression of ROS, pro-inflammatory cytokines (IL-6, TNF-a, and MCP-1) and reduce the activity of MMP-9 in ox-LDL-induced RAW264.7 macrophages (Xu et al., 2011). *Porphyromonas gingivalis(Porphyromonas gingivalis)* is not only a pathogen associated with chronic periodontitis, but also an important risk factor for AS (Bostanci and Belibasakis, 2012). Xuan et al. found that Tan IIA (60 mg/kg/d) could reduce the size of atherosclerotic plaques in ApoE^−/−^ mice induced by *P. gingivalis* infection. Further studies showed that Tan IIA also reduced the expression of CD40, TNF-α, IL-1β, IL-6, and MMP-2 in aortic tissue (Xuan et al., 2017). In another study, Xuan et al. found that Tan IIA (60 mg/kg/d) inhibited the progression of AS in *P. Gingivalis*-induced ApoE^−/−^ mice by down-regulating NOX2, NOX4, and NF-κB (Xuan et al., 2023). Wang et al. found that Tan IIA (90 mg/kg) significantly reduced the size of plaques in the right common carotid artery and increased collagen content and fiber cap thickness in ApoE^−/−^ mice. *In vitro* studies, Tan IIA (50 μmol/L) significantly reduced ox-LDL-induced lipid droplet accumulation in RAW 264.7 macrophages. In addition, Tan IIA downregulates the expression of inflammatory cytokines and MMP-9 by regulating the PI3K/Akt and TLR4/NF-κB signaling pathways (Wang et al., 2020). Previous studies have shown that the receptor of advanced glycation end products (RAGE) signaling pathway is involved in the production of chemokines and adhesion molecules. In addition, RAGE can activate MAPK and NF-κB by binding to ox-LDL or AGE, leading to upregulation of MMP, which accelerates erosion and thinning of the fiber cap and leads to plaque instability (Harja et al., 2008). Zhao et al. found that Tan IIA (30 mg/kg, 8 weeks) can reduce the expression of MMP-2, MMP-3, MMP-9 and inflammatory factors by inhibiting RAGE/MAPK signaling pathway, thus increasing the stability of atherosclerotic plaques (Zhao et al., 2016). (Figure 4)

TLR4/myeloid differentiation factor 88 (MyD88)/NF-κB signaling pathway is one of the important anti-inflammatory and immunomodulatory pathways. Chen et al. found that Tan IIA (10,30 and 90 mg/kg) plays an important role in stabilizing atherosclerotic plaques in HCD-fed ApoE^−/−^ mice by inhibiting the TLR4/MyD88/NF-κB pathway (Chen et al., 2019). Macrophages are the basic components of atherosclerotic plaques and play a key role in plaque instability. It was found that macrophages showed different phenotypes. The M1 phenotype releases pro-inflammatory cytokines and ROS and plays a key role in the initiation of inflammation (Benoit et al., 2008). In contrast, the M2 phenotype produces anti-inflammatory cytokines and is involved in phagocytosis of dead cells (Gordon and Martinez, 2010). Autophagy is a repair process that plays a key role in maintaining cellular homeostasis. Macrophage autophagy can reduce the accumulation of foam cells and inflammation in plaque, and play a role in stabilizing plaque. Chen et al. found that Tan IIA (10 mg/kg) inhibited lipid accumulation in the aorta of HCD-fed ApoE^−/−^ mice and increased the number of M2 phenotypes in AS lesions. *In vitro* studies, Tan IIA (10 μg/μL) enhanced ox-LDL-induced macrophage autophagy and increased M2 phenotype by a mechanism associated with inhibition of the miR-375/KLF4 signaling pathway (Chen et al., 2019). TGF-β is a cytokine involved in cell differentiation, proliferation, growth and apoptosis. Previous studies have found that TGF-β can affect the stability of atherosclerotic plaque by inhibiting monocyte chemotaxis, reducing the proliferation of VSMC, inhibiting ox-LDL absorption, anti-inflammatory and antioxidant (Castañares et al., 2007). Wang et al. found that Tan IIA (10 mg/kg) can reduce inflammatory damage and stabilize atherosclerotic plaque by activating TGF-β/PI3K/Akt/eNOS signaling pathway (Wang et al., 2020). Intracellular chloride channel 1(CLIC1) is a sensor and effector during oxidative stress and plays a major role in diseases involving oxidative stress. Zhu et al. found that STS (10 mg/kg) inhibited oxidative stress and inflammatory response in the aortic atherosclerotic plaques of ApoE^−/−^ mice fed with HCD. *In vitro* studies, STS (0.4 mmol/L) alleviated oxidative stress in HUVEC cells and inhibited the expression of inflammatory factors and adhesion molecules by inhibiting CLIC1 expression and membrane ectopia (Zhu et al., 2017). Cyclooxygenase-2(COX-2) is a well-known inducer of the inflammatory response. Ma et al. found that Tan IIA (25 μg/mL) mitigated the upregulation of inflammatory factors in ox-LDL-induced HUVECs by inhibiting the COX-2/TNF-α/NF-κB signaling pathway (Ma et al., 2023). (Figure 4)

## 6 Tan IIA inhibits lipid deposition

AS is characterized by the deposition of excess cholesterol in the intima of the arteries. Foam cells play a crucial role in the development of AS. CD36 and class A scavenger receptor (SR-A) are important receptors for macrophage uptake of modified lipoproteins, which can increase lipid deposition in macrophages. In addition to cholesterol uptake, the balance of free cholesterol and cholesterol ester is also crucial for the regulation of cholesterol content in macrophages. After internalization, lipoproteins are delivered to lysosomes, where cholesterol ester is hydrolyzed to free cholesterol by lysosomal acid lipase (LAL). To prevent free cholesterol associated cytotoxicity, it is reesterified by Acyl coenzyme A:cholesterol acyltransferase-1 (ACAT1) on the endoplasmic reticulum and stored in cytoplasmic lipid droplets (Zhu et al., 2018). Excess cholesterol ester accumulate in macrophages, leading to the formation of foam cells. Cholesterol ester in the endoplasmic reticulum can be hydrolyzed by neutral cholesteryl ester hydrolase (nCEH) to release free cholesterol for transporter-mediated efflux. ABCA1, ABCG1 and scavenger receptor class B (SR-B) play important roles in macrophage cholesterol expulsion (Zhu et al., 2018). Reverse cholesterol transport (RCT) is described as high-density lipoprotein (HDL) transporting excess cholesterol stored in peripheral tissues to the liver. In the liver, cholesterol can be excreted directly into bile or metabolized into bile acids/salts. Liver X receptor (LXR) is a key transcription factor that regulates cholesterol in macrophages. It drives the expression of efflux genes, including ABCA1 and ABCG1. In addition, LXR can also promote low-density lipoprotein receptor (LDLR) degradation, thereby limiting the uptake of exogenous cholesterol. SREBPs, a membrane-bound transcription factor, regulates the expression of genes associated with cholesterol intake, such as LDLR (Zhu et al., 2018).

Ox-LDL induces expression of scavenger receptors, including CD36 and SR-A, in macrophages. Studies have shown that PPARγ is involved in the regulation of CD36 by ox-LDL (Nagy et al., 1998). Tang et al. found that Tan IIA (10–90 mg/kg) can reduce the atherosclerotic plaques in the aortic arch of ApoE^−/−^ mice, and reduce the expression of CD36 and SR-A mRNA in the aorta. *In vitro* studies, Tan IIA (0.1–10 μM) reduced mRNA and protein expression of CD36 in ox-LDL-induced macrophages, but had no effect on SR-A expression, the mechanism of which is related to inhibition of PPARγ (Tang et al., 2011). Jia et al. found that Tan IIA (10 mg/kg) increased LDLR and SREBP-2 protein expression in the liver tissue of HCD-fed SD rats and significantly reduced hepatic lipid deposition. In addition, Tan IIA increased the expression of ABCA1 protein and decreased the expression of CD36 in macrophages (Jia et al., 2016). Previous studies have shown that elevated levels of ox-LDL in serum are one of the risk factors for AS (Anselmi et al., 2006). Chen et al. found that Tan IIA (3,10 and 30 mg/kg/d) could reduce the ox-LDL content in serum and aorta of HCD-fed New Zealand white rabbits, and increase the activity of SOD and glutathione peroxidase (GPx), thereby improving AS (Chen et al., 2012). HO-1, an anti-infection and antioxidant enzyme, is considered as a new target for the treatment of AS (Stocker and Perrella, 2006). Liu et al. found that Tan IIA (30 mg/kg/d) reduced the expression of SR-A and CD36 and increased the expression of ABCA1 and ABCG1 in the aorta of ApoE^−/−^ mice, thereby shrinking atherosclerotic plaques. *In vitro* studies, Tan IIA (1,3 and 10 μM) downregulated the expression of SR-A and upregulated the expression of ABCA1 and ABCG1 by activating the ERK/Nrf2/HO-1 signaling pathway, thereby inhibiting ox-LDL-induced cholesterol accumulation in THP-1 macrophages (Liu et al., 2014). Omentin-1 is a beneficial adipokine that has a preventive effect on AS (Watanabe et al., 2016). Tan et al. found that Tan IIA (20, 40 and 80 mg/L) inhibited excessive cholesterol accumulation in THP-1 macrophages by upregulating the Omentin-1/ABCA1 pathway (Tan et al., 2019). (Figure 4)

Macrophage efferocytosis refers to the process of phagocytosis and degradation of apoptotic cells by macrophages (Jia et al., 2022). Effective efferocytosis can remove necrotic and dead cells from the lesion, reduce tissue damage, and also reduce AS (Gerlach et al., 2021). Impaired efferocytosis function may further accelerate the progression of AS (Kasikara et al., 2021). Efficient efferocytosis can effectively degrade lipids swallowed by macrophages and reduce the production of foam cells. Wang et al. found that Tan IIA (intraperitoneal injection, 15 mg/kg/d) enhanced the expression of efferocytosis-related signals and the clearance of apoptotic cells in the aorta of LDLR^−/−^ mice fed a high-fat diet. Efficient efferocytosis can also effectively degrade lipids swallowed by macrophages and reduce the production of foam cells. *In vitro* studies, Wang et al. found that Tan IIA (20 or 40 μM/L) increased the expression of efferocytosis-related molecules in ox-LDL-induced RAW264.7 macrophages and reduced the accumulation of macrophage-derived foam cells (Wang et al., 2023). (Figure 4)

Autophagy has been shown to be involved in lipid metabolism (Kovsan et al., 2010). During the development of AS, the activation of macrophage autophagy contributes to the breakdown of intracellular lipids, thereby reducing foam cell formation (Ouimet et al., 2011). Qian et al. found that Tan IIA (10 mg/kg/d) can reduce lipid accumulation in atherosclerotic plaques of ApoE^−/−^ mice fed high fat diet by up-regulating autophagy. *In vitro* studies, Tan IIA (10 μg/μL) can inhibit ox-LDL-induced lipid deposition in RAW264.7 macrophages by inhibiting miR-214-3p/autophagy-related protein-16-like protein 1 (ATG16L1) pathway to promote autophagy (Qian et al., 2023). (Figure 4)

## 7 Tan IIA inhibits monocyte adhesion to vascular endothelial cells

Adhesion molecules play an important role in the formation of AS. Adhesion molecules associated with AS include immunoglobulin superfamily (IgSF), selectin family and integrin family. IgSF can interact with integrin family adhesion molecules to participate in cell recognition and adhesion. There are three main types of IgSF: platelet endothelial cell adhesion molecule-1 (PECAM-1), ICAM-1 and VCAM-1. The selectin family is mainly involved in rolling adhesion of leukocytes. There are three types of this family: P-selectin, e-selectin, and leukocyte selectin (L-selectin). The integrin family is a glycoprotein receptor that exists on the surface of cells (Zhu et al., 2018). It is well known that AS is an inflammatory process (Libby, 2002). The initial process of inflammation is the migration of white blood cells in the vascular lumen and their adherence to VECs. Cell adhesion molecules are critical in the adhesion of circulating monocytes to VECs (Qian et al., 2012).

Previous studies have shown that VCAM-1 promoters contain several transcription factor binding sites, including NF-κB, GATAs, specificity protein-1(SP-1), AP-1, and interferon regulatory factor-1(IRF-1) binding site. Nizamutdinova et al. found that Tan IIA (1, 5, 10 and 50 μM) inhibited VCAM-1 secretion by TNF-α-induced HUVEC cells. The mechanism is related to the downregulation of IRF-1 and GATA transcription factors by inhibiting PI3/Akt, protein kinase C(PKC) and JAK/STAT3 signaling pathways (Nizamutdinova et al., 2012). Fractalkine is an atypical chemokine that binds to CX3C chemokine receptor 1(CX3CR1) in nearby cells to induce cellular chemotaxis (Bazan et al., 1997). Therefore, fractalkine plays a key role in nearby monocyte homeostasis in the late stages of AS (Stolla et al., 2012). Chang et al. found that Tan IIA (1, 5, 10 and 20 μM) significantly inhibited the adhesion of TNF-α-induced THP-1 monocytes to HUVEC, and the mechanism was related to the inhibition of VCAM-1, ICAM-1 and fractalkine (Chang et al., 2014). EPCs are a type of cell involved in endothelial repair. EPCs proliferates in the bone marrow and is released upon vascular injury, migrates to the injury site and matures into VECs (Altabas et al., 2016). Monocytes can adhere to circulating EPCs via VCAM-1 and ICAM-1. When EPCs are incorporated into VECs, these monocytes are implanted into damaged blood vessels. Yang et al. found that Tan IIA (1, 5, 10 and 20 μM) can downregulate VCAM-1 and ICAM-1 by inhibiting the NF-κB pathway, thereby reducing the adhesion of monocytes to EPCs (Yang et al., 2016). In another study, Tang et al. found that Tan IIA (5, 10 and 20 μM) downregulates VCAM-1 and ICAM-1 expression by inhibiting NF-κB activation and ROS production in BMVECs, thereby inhibiting TNF-α-induced monocyte adhesion to BMVEC (Tang et al., 2011). Pentraxin 3(PTX3), an acute phase protein, can be induced by a variety of pro-atherosclerotic stimuli, such as LPS, TNF-α, IL-1β, and high sugar levels. PTX3 is closely related to a variety of CVDs. For example, PTX3 can damage the production of vasoactive NO in VECs and promote the formation of foam cells (Liu et al., 2014; Carrizzo et al., 2015). Fang et al. found that Tan IIA (0.1–5 μM) can reduce the expression of PTX3 in TNF-α-induced HUVECs and inhibit the adhesion of THP-1 monocytes to endothelial cells by inhibiting the p38 MAPK/NF-κB signaling pathway (Fang et al., 2018). (Figure 4)

## 8 Pharmacokinetics and toxicology of Tan ⅡA

Most of the pharmacokinetics and safety reports of Tan ⅡA came from Chinese literature. Tan ⅡA is insoluble in water, and its oral preparation has poor intestinal absorption and low bioavailability (Li et al., 2014). Its absorption mechanism in the stomach may be passive transport, while its absorption mechanism in the intestine may be active diffusion, and the colon is the best site for absorption. It has been reported that there are a variety of bacteria and bioconvertases in the colon, which also promote the absorption of Tan ⅡA (Hu et al., 2008). Hao et al. studied the tissue distribution of Tan ⅡA in rats. They injected 2 mg/kg of TanⅡA intravenously into rats, and after 15 min, TanⅡA rapidly distributed to various tissues, and the concentration of Tan ⅡA in liver, lung, heart, kidney and brain tissues decreased successively. Lower concentrations in brain tissue indicate a poor ability to cross the blood-brain barrier. In addition, the distribution of Tan ⅡA in the kidney was found to be relatively low (Hao et al., 2006). Zhao et al. gave 60 healthy subjects a single intramuscular injection of STS 40mg and 80 mg respectively, and then measured the drug concentration of STS injection in plasma. The results showed that the half-life of the drug was 52.5–63.6 min, and the peak time was 41.9–56.7 min (Zhao et al., 2023). Bi et al. studied the metabolism of Tan ⅡA in rat liver microsomal enzymes. The results showed that the maximum velocity (Vmax), kinetics parameters michaelis constant (Km) and intrinsic clearance (CLint) of Tan ⅡA in rat liver microsomal enzyme were (1.20 ± 0.18) nmol·min^-1^·mg, (4.35 ± 0.67) nmol/mL, and (0.28 ± 0.06) mL·min^-1^·mg. In a follow-up experiment, Bi et al. observed the effects of different types of cytochrome P450(CYP) enzyme specific inhibitors on TanⅡA metabolism. They found that CYP2C19 and CYP3A1 are mainly involved in the metabolism of Tan ⅡA, and CYP2D6 also plays a part in the metabolism (Bi et al., 2007).

STS is a water-soluble substance obtained by sulfonation of Tan ⅡA. Compared with other traditional Chinese medicine injections prepared from *Salvia miltiorrhiza* extract, the adverse drug reactions (ADRs) were less and the safety was higher. Wang et al. (Wang et al., 2014) comprehensively analyzed 27 adverse events of STS injection according to clinical case reports and literature. The results showed that the main adverse reactions of STS were allergic reactions (about 30%), which mainly involved skin and its appendages, including skin rash, skin itching and local reaction after injection. The systemic damage and circulatory system reaction of STS were mainly manifested as anaphylactic shock, chills, fever, chest tightness and blood pressure drop. After symptomatic treatment, the adverse reactions of STS were alleviated or disappeared, and there was little effect on the original disease and no obvious sequelae. Although no death cases were reported in the collected literature, there were 6 cases of life-threatening anaphylactic shock, which suggested that the serious adverse reactions of TanⅡA should be closely monitored and timely treatment to avoid serious consequences. TanⅡA is an extract of *S. miltiorrhiza*, which has the characteristics of complex composition and insufficient purity of effective monomer in the process of processing and extraction, which is easy to cause the production of allergic media. In addition, STS may precipitate insoluble TanⅡA sulfonic acid during drug storage and use, resulting in particles entering the body, which can easily lead to allergic reactions (Shi et al., 2007). Shi et al. conducted a safety-related analysis of STS injection in patients at Zhongshan Hospital Affiliated to Fudan University. Of the 125 patients using STS injection, no serious adverse events occurred, and 7 (5.6%) had mild adverse events. These mild adverse events included 4 cases of abnormal elevated liver aminotransferase levels, 1 case of abnormal elevated aminotransferase levels with suspected positive fecal occult blood, 1 case of abnormal elevated aminotransferase levels with ecchymosis on the right forearm, and 1 case of left eye conjunctival hemorrhage. All seven patients were treated with other drugs that may cause these adverse events, such as statins and antithrombotic drugs, during the STS injection. TanⅡA and statins are metabolized mainly by P450 enzymes, which are important enzymes involved in drug metabolism in the body and can be competed or inhibited. Shi et al. suspected that STS injection may affect the metabolic rate of statins and increase their blood concentration, which may lead to an abnormal increase in the level of transaminase (Shi et al., 2016). Most ADRs of TanⅡA occur during the first medication cycle. More than one-third of ADRs occurred within 40 min, but 17.4% of ADRs occurred more than 1 day (Shu et al., 2015). Before using STS, medical staff should understand the patient’s allergy history, liver and kidney function, and underlying diseases in detail, and make a comprehensive assessment to decide whether to use STS. Finally, first aid preparations should be made in advance to reduce the occurrence of adverse reactions.

## 9 Discussion

Tan IIA, a fat-soluble compound extracted from the Chinese herb *S. miltiorrhiza*, has a protective effect on blood vessels. This paper reviews the mechanism of Tan IIA in protecting blood vessels. Tan IIA can reduce VECs damage by inhibiting oxidative stress and inflammation. In addition, Tan IIA can also improve endothelium-dependent vasodilation function and protect the vascular endothelial barrier. Tan IIA can also reduce vascular stenosis by inhibiting inflammation, proliferation, and migration of VSMC cells, and improve the stability of the fibrous cap of atherosclerotic plaque by inhibiting VSMC apoptosis. Finally, Tan IIA can inhibit inflammatory response and lipid deposition in atherosclerotic plaques, inhibit MC adhesion to VECs and inhibit platelet activation.

The protective mechanism of Tan IIA on blood vessels is complex and networked. At present, researchers have not studied the pharmacological mechanism of Tan IIA deeply. The pathway identified in previous studies may only be part of the downstream signaling pathway after Tan IIA acts on a specific molecular target. Therefore, we propose to investigate specific molecular targets of Tan IIA using a systems biology approach. For example, Ma et al. used a photosensitive probe to label metformin and eventually identified PEN2 as a direct target of metformin (Ma et al., 2022).

It should be noted that the results from different studies are not entirely consistent. Wang et al. (Wang et al., 2020) found that Tan IIA (90 mg/kg) significantly reduced the size of the right common carotid atherosclerotic plaque in ApoE^−/−^ mice, while Zhao et al. (Zhao et al., 2016) found that, Tan IIA (30 mg/kg) did not change atherosclerotic plaque size in ApoE^−/−^ mice. The two studies reached inconsistent conclusions, which may be related to the different doses of Tan IIA. Tang et al. (Tang et al., 2011) found that Tan IIA (0.1–10μM, 24 h) reduced the protein expression of CD36 in ox-LDL (50 μg/mL, 24 h)-induced macrophages, but had no effect on the expression of SR-A. However, Liu et al. (Liu et al., 2014) found that Tan IIA (1, 3 and 10 μM, 24 h) could inhibit ox-LDL (10 μg/mL, 4 h)-induced cholesterol accumulation in THP-1 macrophages by down-regulating SR-A expression. The regulation of Tan IIA on SR-A expression was inconsistent between the two studies, which may be related to ox-LDL concentration and intervention time.

Tan ⅡA is insoluble in water, poor intestinal absorption of oral preparations, and low bioavailability, which limits the clinical use of TanⅡA. STS is a water-soluble substance obtained by sulfonation of Tan ⅡA, which improves bioavailability. It should be pointed out that STS has some adverse reactions when used in some patients. The main adverse reaction of STS is anaphylaxis (about 30%), which mainly involves the skin and its appendages. Although the adverse effects of STS are mostly mild, some life-threatening conditions can occur, such as anaphylactic shock. Before using STS, medical personnel should ask patients about their allergy history, liver and kidney function, and underlying diseases to comprehensively evaluate whether STS can be used.

Finally, the effect of Tan ⅡA in AS is mostly studied in animal models and cell models, and there are few clinical trial data. Therefore, we suggest that large-scale clinical trials should be conducted to evaluate the efficacy of TanⅡA in protecting against AS.

## 10 Conclusion

In conclusion, the mechanism by which Tan II A protects blood vessels is complex. It should be noted that the pharmacological mechanism of Tan II A has been studied mainly through animal and cell experiments, and there are few clinical studies. In addition, we need to pay attention to the adverse reactions of Tan IIA. Before using STS injection, clinical staff should know the patient’s allergy history, liver and kidney function and underlying diseases in detail. Finally, we suggest that researchers use systems biology approaches to identify specific molecular targets for Tan IIA and fundamentally elucidate the mechanism of Tan IIA.

## Figures and Tables

**Table:** 

**AS**	atherosclerosis
**AMI**	acute myocardial infarction
**CVDs**	cardiovascular diseases
**Tan ⅡA**	Tanshinone ⅡA
**VSMC**	vascular smooth muscle cells
**VECs**	vascular endothelial cells
**ECD**	endothelial cell dysfunction
**H2O2**	Hydrogen peroxide
**SOD**	superoxide dismutase
**CD40**	cluster of differentiation 40
**ATF**	activated transcription factor
**HUVECs**	human umbilical vein endothelial cells
**PXR**	pregnane X receptor
**H2S**	Hydrogen sulfide
**CSE**	cystethionine gamma-lyase
**ER**	estrogen receptor
**cAMP**	cyclic adenosine monophosphate
**Nrf2**	nuclear factor erythroid 2-related factor 2
**HCAECs**	human coronary artery endothelial cells
**eNOS**	Endothelial nitric oxide synthase
**PP2A**	protein phosphatase 2A
**BH4**	tetrahydrobiopterin
**BH2**	dihydrobiopterin
**NADPH**	nicotinamide-adenine dinucleotide phosphate
**NOX4**	NADPH oxidase 4
**HSP90**	heat shock protein 90
**CMECs**	cardiac microvascular endothelial cells
**ERK1/2**	extracellular signalregulated kinase 1/2
**ET-1**	endothelin-1
**ETA**	endothelin-A receptor
**ETB**	endothelin-B receptor
**ECE-1**	endothelin-converting enzyme-1
**TNF-α**	tumor necrosis factor-α
**BMVECs**	brain microvascular endothelial cells
**OSAHS**	obstructive sleep apnea-hypopnea syndrome
**BMP9**	bone morphogenetic protein 9
**BMPR2**	bone morphogenetic protein receptor type 2
**Smad1/5/9**	mothers against decapentaplegic homolog 1/5/9
**LPS**	Lipopolysaccharide
**EPCs**	endothelial progenitor cells
**DM**	diabetes mellitus
**MGO**	Methylglyoxal
**AGEs**	advanced glycation end products
**HBMEC**	human brain microvascular endothelial cells
**MAPK**	mitogen-activated protein kinase
**HHcy**	Hyperhomocysteinemia
**Hcy**	homocysteine
**NNMT**	nicotinamide N-methyltransferase
**Sirt1**	sirtuin1
**NLRP3**	NLR family pyrin domain containing 3
**CC**	cholesterol crystals
**AMPK**	Adenosine 5‘-monophosphate-activated protein kinase
**ECM**	extracellular matrix
**PCI**	percutaneous coronary intervention
**RAAS**	renin-angiotensin-aldosterone system
**MMP2**	matrix metalloproteinase 2
**miRNA**	microRNA
**TPM1**	tropomyosin 1
**HASMC**	human aortic smooth muscle cell
**TRPC3**	Transient receptor potential cation channel subfamily C member 3
**PDK1**	3’-phosphoinostitide dependent kinase
**AP-1**	activator protein-1
**TLR4**	toll-like receptors 4
**TGF-β**	transforming growth factor-β
**TAK1**	TGF-β-activated kinase 1
**KLF4**	Krüppel-like factor 4
**PDGF**	platelet-derived growth factor
**iNOS**	inducible nitric oxide synthase
**ICAM-1**	intracellular adhesion molecule-1
**VCAM-1**	vascular cell adhesion molecule-1
**P-selectin**	platelet selectin
**e-selectin**	endothelial selectin
**LOX-1**	lectin-like ox-LDL receptor-1
**CD36**	cluster of differentiation 36
**BMDMs**	bone marrow-derived macrophages
**HCD**	high cholesterol diet
**Aβ**	Amyloid β peptide
**APP**	amyloid precursor protein
**CircRNA**	Circular RNA
**lncRNA**	long non-coding RNA
**mRNA**	messenger RNA
**RAGE**	receptor of advanced glycation end products
**MyD88**	myeloid differentiation factor 88
**CLIC1**	intracellular chloride channel 1
**COX-2**	Cyclooxygenase-2
**SR-A**	class A scavenger receptor
**LAL**	lysosomal acid lipase
**ACAT1**	Acyl coenzyme A:cholesterol acyltransferase-1
**nCEH**	neutral cholesteryl ester hydrolase
**RCT**	Reverse cholesterol transport
**HDL**	high-density lipoprotein
**LXR**	Liver X receptor
**LDLR**	low-density lipoprotein receptor
**GPx**	glutathione peroxidase
**IgSF**	immunoglobulin superfamily
**PECAM-1**	platelet endothelial cell adhesion molecule-1
**L-selectin**	leukocyte selectin
**SP-1**	specificity protein-1
**IRF-1**	interferon regulatory factor-1
**PKC**	protein kinase C
**CX3CR1**	CX3C chemokine receptor 1
**PTX3**	Pentraxin 3
**CYP**	cytochrome P450
**ADRs**	adverse drug reactions

## References

[B1] Ackers I. Szymanski C. Duckett K. J. Consitt L. A. Silver M. J. Malgor R. (2018). Blocking Wnt5a signaling decreases CD36 expression and foam cell formation in atherosclerosis. Cardiovasc Pathol. 34, 1–8. 10.1016/j.carpath.2018.01.008 29474941

[B2] Alexander M. R. Owens G. K. (2012). Epigenetic control of smooth muscle cell differentiation and phenotypic switching in vascular development and disease. Annu. Rev. Physiol. 74, 13–40. 10.1146/annurev-physiol-012110-142315 22017177

[B3] Altabas V. Altabas K. Kirigin L. (2016). Endothelial progenitor cells (EPCs) in ageing and age-related diseases: how currently available treatment modalities affect EPC biology, atherosclerosis, and cardiovascular outcomes. Mech. Ageing Dev. 159, 49–62. 10.1016/j.mad.2016.02.009 26919825

[B4] Anselmi M. Garbin U. Agostoni P. Fusaro M. Pasini A. F. Nava C. (2006). Plasma levels of oxidized-low-density lipoproteins are higher in patients with unstable angina and correlated with angiographic coronary complex plaques. Atherosclerosis 185 (1), 114–120. 10.1016/j.atherosclerosis.2005.05.020 15998517

[B5] Bazan J. F. Bacon K. B. Hardiman G. Wang W. Soo K. Rossi D. (1997). A new class of membrane-bound chemokine with a CX3C motif. Nature 385 (6617), 640–644. 10.1038/385640a0 9024663

[B6] Bennett M. R. Sinha S. Owens G. K. (2016). Vascular smooth muscle cells in atherosclerosis. Circ. Res. 118 (4), 692–702. 10.1161/CIRCRESAHA.115.306361 26892967 PMC4762053

[B7] Benoit M. Desnues B. Mege J. L. (2008). Macrophage polarization in bacterial infections. J. Immunol. 181 (6), 3733–3739. 10.4049/jimmunol.181.6.3733 18768823

[B8] Berezin A. E. Kremzer A. A. Berezina T. A. Martovitskaya Y. V. (2016). The pattern of circulating microparticles in patients with diabetes mellitus with asymptomatic atherosclerosis. Acta Clin. Belg 71 (1), 38–45. 10.1080/17843286.2015.1110894 27075791

[B9] Bergmann K. Sypniewska G. (2014). Secreted frizzled-related protein 4 (SFRP4) and fractalkine (CX3CL1) - potential new biomarkers for β-cell dysfunction and diabetes. Clin. Biochem. 47 (7-8), 529–532. 10.1016/j.clinbiochem.2014.03.007 24675103

[B10] Bhatt P. M. Malgor R. (2014). Wnt5a: a player in the pathogenesis of atherosclerosis and other inflammatory disorders. Atherosclerosis 237 (1), 155–162. 10.1016/j.atherosclerosis.2014.08.027 25240110 PMC4252768

[B11] Bi H. C. He F. Wen Y. Y. Chen X. Huang M. (2007). Metabolic kinetics of tanshinone IIA in rat liver microsomal enzymes. Chin. Herb. Med. 6, 882–886.

[B12] Bostanci N. Belibasakis G. N. (2012). Porphyromonas gingivalis: an invasive and evasive opportunistic oral pathogen. FEMS Microbiol. Lett. 333 (1), 1–9. 10.1111/j.1574-6968.2012.02579.x 22530835

[B13] Campbell J. H. Campbell G. R. (1986). Endothelial cell influences on vascular smooth muscle phenotype. Annu. Rev. Physiol. 48, 295–306. 10.1146/annurev.ph.48.030186.001455 3518616

[B14] Carrizzo A. Lenzi P. Procaccini C. Damato A. Biagioni F. Ambrosio M. (2015). Pentraxin 3 induces vascular endothelial dysfunction through a P-selectin/Matrix metalloproteinase-1 pathway. Circulation 131 (17), 1495–1505. ; discussion 1505. 10.1161/CIRCULATIONAHA.114.014822 25747934

[B15] Castañares C. Redondo-Horcajo M. Magán-Marchal N. ten Dijke P. Lamas S. Rodríguez-Pascual F. (2007). Signaling by ALK5 mediates TGF-beta-induced ET-1 expression in endothelial cells: a role for migration and proliferation. J. Cell Sci. 120 (Pt 7), 1256–1266. 10.1242/jcs.03419 17376964

[B16] Chan P. Chen Y. C. Lin L. J. Cheng T. H. Anzai K. Cheng Y. H. (2012). Tanshinone IIA Attenuates H₂O₂ -induced injury in human umbilical vein endothelial cells. Am. J. Chin. Med. 40 (6), 1307–1319. 10.1142/S0192415X12500966 23227799

[B17] Chang C. C. Chu C. F. Wang C. N. Wu H. T. Bi K. W. Pang J. H. S. (2014). The anti-atherosclerotic effect of tanshinone IIA is associated with the inhibition of TNF-α-induced VCAM-1, ICAM-1 and CX3CL1 expression. Phytomedicine 21 (3), 207–216. 10.1016/j.phymed.2013.09.012 24157079

[B18] Chen L. Guo Q. H. Chang Y. Zhao Y. S. Ji E. S. (2017). Tanshinone IIA ameliorated endothelial dysfunction in rats with chronic intermittent hypoxia. Cardiovasc Pathol. 31, 47–53. 10.1016/j.carpath.2017.06.008 28985491

[B19] Chen W. Li X. Guo S. Song N. Wang J. Jia L. (2019b). Tanshinone IIA harmonizes the crosstalk of autophagy and polarization in macrophages via miR-375/KLF4 pathway to attenuate atherosclerosis. Int. Immunopharmacol. 70, 486–497. 10.1016/j.intimp.2019.02.054 30870679

[B20] Chen W. Tang F. Xie B. Chen S. Huang H. Liu P. (2012). Amelioration of atherosclerosis by tanshinone IIA in hyperlipidemic rabbits through attenuation of oxidative stress. Eur. J. Pharmacol. 674 (2-3), 359–364. 10.1016/j.ejphar.2011.10.040 22088276

[B21] Chen Z. Gao X. Jiao Y. Qiu Y. Wang A. Yu M. (2019a). Tanshinone IIA exerts anti-inflammatory and immune-regulating effects on vulnerable atherosclerotic plaque partially *via* the TLR4/MyD88/NF-κB signal pathway. Front. Pharmacol. 10, 850. 10.3389/fphar.2019.00850 31402870 PMC6677033

[B22] Cheng J. Chen T. Li P. Wen J. Pang N. Zhang L. (2018). Sodium tanshinone IIA sulfonate prevents lipopolysaccharide-induced inflammation via suppressing nuclear factor-κB signaling pathway in human umbilical vein endothelial cells. Can. J. Physiol. Pharmacol. 96 (1), 26–31. 10.1139/cjpp-2017-0023 28658584

[B23] Chistiakov D. A. Melnichenko A. A. Grechko A. V. Myasoedova V. A. Orekhov A. N. (2018). Potential of anti-inflammatory agents for treatment of atherosclerosis. Exp. Mol. Pathol. 104 (2), 114–124. 10.1016/j.yexmp.2018.01.008 29378168

[B24] Clarke M. C. Figg N. Maguire J. J. Davenport A. P. Goddard M. Littlewood T. D. (2006). Apoptosis of vascular smooth muscle cells induces features of plaque vulnerability in atherosclerosis. Nat. Med. 12 (9), 1075–1080. 10.1038/nm1459 16892061

[B25] Clarke M. C. Talib S. Figg N. L. Bennett M. R. (2010). Vascular smooth muscle cell apoptosis induces interleukin-1-directed inflammation: effects of hyperlipidemia-mediated inhibition of phagocytosis. Circ. Res. 106 (2), 363–372. 10.1161/CIRCRESAHA.109.208389 19926874

[B26] Clozel M. Gray G. A. Breu V. Löffler B. M. Osterwalder R. (1992). The endothelin ETB receptor mediates both vasodilation and vasoconstriction *in vivo* . Biochem. Biophys. Res. Commun. 186 (2), 867–873. 10.1016/0006-291x(92)90826-7 1323294

[B27] Dixon S. J. Lemberg K. M. Lamprecht M. R. Skouta R. Zaitsev E. M. Gleason C. E. (2012). Ferroptosis: an iron-dependent form of nonapoptotic cell death. Cell 149 (5), 1060–1072. 10.1016/j.cell.2012.03.042 22632970 PMC3367386

[B28] Fan G. Zhu Y. Guo H. Wang X. Wang H. Gao X. (2011). Direct vasorelaxation by a novel phytoestrogen tanshinone IIA is mediated by nongenomic action of estrogen receptor through endothelial nitric oxide synthase activation and calcium mobilization. J. Cardiovasc Pharmacol. 57 (3), 340–347. 10.1097/FJC.0b013e31820a0da1 21383591

[B29] Fan G. W. Gao X. M. Wang H. Zhu Y. Zhang J. Hu L. M. (2009). The anti-inflammatory activities of Tanshinone IIA, an active component of TCM, are mediated by estrogen receptor activation and inhibition of iNOS. J. Steroid Biochem. Mol. Biol. 113 (3-5), 275–280. 10.1016/j.jsbmb.2009.01.011 19429433

[B30] Fang J. Chen Q. He B. Cai J. Yao Y. Cai Y. (2018). Tanshinone IIA attenuates TNF-α induced PTX3 expression and monocyte adhesion to endothelial cells through the p38/NF-κB pathway. Food Chem. Toxicol. 121, 622–630. 10.1016/j.fct.2018.09.063 30268796

[B31] Förstermann U. (2010). Nitric oxide and oxidative stress in vascular disease. Pflugers Arch. 459 (6), 923–939. 10.1007/s00424-010-0808-2 20306272

[B32] Gerlach B. D. Ampomah P. B. Yurdagul A. Jr Liu C. Lauring M. C. Wang X. (2021). Efferocytosis induces macrophage proliferation to help resolve tissue injury. Cell Metab. 33 (12), 2445–2463.e8. 10.1016/j.cmet.2021.10.015 34784501 PMC8665147

[B33] Gimbrone M. A. Jr García-Cardeña G. (2016). Endothelial cell dysfunction and the pathobiology of atherosclerosis. Circ. Res. 118 (4), 620–636. 10.1161/CIRCRESAHA.115.306301 26892962 PMC4762052

[B34] Gordon S. Martinez F. O. (2010). Alternative activation of macrophages: mechanism and functions. Immunity 32 (5), 593–604. 10.1016/j.immuni.2010.05.007 20510870

[B35] Hao H. Wang G. Cui N. Li J. Xie L. Ding Z. (2006). Pharmacokinetics, absorption and tissue distribution of tanshinone IIA solid dispersion. Planta Med. 72 (14), 1311–1317. 10.1055/s-2006-951698 17024606

[B36] Harja E. Bu D. X. Hudson B. I. Chang J. S. Shen X. Hallam K. (2008). Vascular and inflammatory stresses mediate atherosclerosis via RAGE and its ligands in apoE-/- mice. J. Clin. Invest. 118 (1), 183–194. 10.1172/JCI32703 18079965 PMC2129235

[B37] Hartmann P. Zhou Z. Natarelli L. Wei Y. Nazari-Jahantigh M. Zhu M. (2016). Endothelial Dicer promotes atherosclerosis and vascular inflammation by miRNA-103-mediated suppression of KLF4. Nat. Commun. 7, 10521. 10.1038/ncomms10521 26837267 PMC4742841

[B38] Hashimoto Y. Zhang C. Kawauchi J. Imoto I. Adachi M. T. Inazawa J. (2002). An alternatively spliced isoform of transcriptional repressor ATF3 and its induction by stress stimuli. Nucleic Acids Res. 30 (11), 2398–2406. 10.1093/nar/30.11.2398 12034827 PMC117192

[B39] He L. Liu Y. Y. Wang K. Li C. Zhang W. Li Z. Z. (2021). Tanshinone IIA protects human coronary artery endothelial cells from ferroptosis by activating the NRF2 pathway. Biochem. Biophys. Res. Commun. 575, 1–7. 10.1016/j.bbrc.2021.08.067 34454174

[B40] Higashi Y. Noma K. Yoshizumi M. Kihara Y. (2009). Endothelial function and oxidative stress in cardiovascular diseases. Circ. J. 73 (3), 411–418. 10.1253/circj.cj-08-1102 19194043

[B41] Hisa I. Kaji H. Inoue Y. Sugimoto T. Chihara K. (2008). Fasting plasma glucose levels are related to bone mineral density in postmenopausal women with primary hyperparathyroidism. Int. J. Clin. Exp. Med. 1 (4), 319–326. 19079676 PMC2596330

[B42] Hong H. J. Hsu F. L. Tsai S. C. Lin C. H. Liu J. C. Chen J. J. (2012). Tanshinone IIA attenuates cyclic strain-induced endothelin-1 expression in human umbilical vein endothelial cells. Clin. Exp. Pharmacol. Physiol. 39 (1), 63–68. 10.1111/j.1440-1681.2011.05637.x 22032308

[B43] Hu X. D. Zhao X. L. Gao C. F. Zhang L. L. Chen D. W. (2008). Absorption kinetics of total ketone of salvia miltiorrhiza on gastrointestinal tract in rats. J. Shenyang Pharm. Univ. 11, 856–859. 10.14066/j.cnki.cn21-1349/r.2008.11.001

[B44] Inoue T. Node K. (2009). Molecular basis of restenosis and novel issues of drug-eluting stents. Circ. J. 73 (4), 615–621. 10.1253/circj.cj-09-0059 19282604

[B45] Jia D. Chen S. Bai P. Luo C. Liu J. Sun A. (2022). Cardiac resident macrophage-derived legumain improves cardiac repair by promoting clearance and degradation of apoptotic cardiomyocytes after myocardial infarction. Circulation 145 (20), 1542–1556. 10.1161/CIRCULATIONAHA.121.057549 35430895

[B46] Jia L. Q. Yang G. L. Ren L. Chen W. N. Feng J. Y. Cao Y. (2012). Tanshinone IIA reduces apoptosis induced by hydrogen peroxide in the human endothelium-derived EA.hy926 cells. J. Ethnopharmacol. 143 (1), 100–108. 10.1016/j.jep.2012.06.007 22750433

[B47] Jia L. Q. Zhang N. Xu Y. Chen W. n. Zhu M. l. Song N. (2016). Tanshinone IIA affects the HDL subfractions distribution not serum lipid levels: involving in intake and efflux of cholesterol. Arch. Biochem. Biophys. 592, 50–59. 10.1016/j.abb.2016.01.001 26820219

[B48] Jia S. Ma W. D. Zhang C. Y. Zhang Y. Yao Z. H. Quan X. H. (2019). Tanshinone IIA attenuates high glucose induced human VSMC proliferation and migration through miR-21-5p-mediated tropomyosin 1 downregulation. Arch. Biochem. Biophys. 677, 108154. 10.1016/j.abb.2019.108154 31672498

[B49] Jiao Y. Li S. M. Gao Z. Y. (2013). etc. Research progress of sodium tanshinone ⅡA sulfonate injection and inflammatory factors in coronary heart disease. World Traditional Chin. Med. 12, 1404–1406. 10.3969/j.Issn.1673-7202.2013.12.007

[B50] Jin U. H. Suh S. J. Chang H. W. Son J. K. Lee S. H. Son K. H. (2008). Tanshinone IIA from Salvia miltiorrhiza BUNGE inhibits human aortic smooth muscle cell migration and MMP-9 activity through AKT signaling pathway. J. Cell Biochem. 104 (1), 15–26. 10.1002/jcb.21599 17979138

[B51] Jin X. Feng C. Y. Xiang Z. Chen Y. P. Li Y. M. (2016). CircRNA expression pattern and circRNA-miRNA-mRNA network in the pathogenesis of nonalcoholic steatohepatitis. Oncotarget 7 (41), 66455–66467. 10.18632/oncotarget.12186 27677588 PMC5341813

[B52] Kasikara C. Schilperoort M. Gerlach B. Xue C. Wang X. Zheng Z. (2021). Deficiency of macrophage PHACTR1 impairs efferocytosis and promotes atherosclerotic plaque necrosis. J. Clin. Invest. 131 (8), e145275. 10.1172/JCI145275 33630758 PMC8262505

[B53] Kokjohn T. A. Van Vickle G. D. Maarouf C. L. Kalback W. M. Hunter J. M. Daugs I. D. (2011). Chemical characterization of pro-inflammatory amyloid-beta peptides in human atherosclerotic lesions and platelets. Biochim. Biophys. Acta 1812 (11), 1508–1514. 10.1016/j.bbadis.2011.07.004 21784149 PMC3185199

[B54] Kovsan J. Bashan N. Greenberg A. S. Rudich A. (2010). Potential role of autophagy in modulation of lipid metabolism. Am. J. Physiol. Endocrinol. Metab. 298 (1), E1–E7. 10.1152/ajpendo.00562.2009 19887596

[B55] Lan J. Li K. Gresham A. Miao J. (2022). Tanshinone IIA sodium sulfonate attenuates inflammation by upregulating circ-Sirt1 and inhibiting the entry of NF-κB into the nucleus. Eur. J. Pharmacol. 914, 174693. 10.1016/j.ejphar.2021.174693 34896110

[B56] Latz E. Xiao T. S. Stutz A. (2013). Activation and regulation of the inflammasomes. Nat. Rev. Immunol. 13 (6), 397–411. 10.1038/nri3452 23702978 PMC3807999

[B57] Li F. Q. Zeng D. K. Jia C. L. Zhou P. Yin L. Zhang B. (2015b). The effects of sodium tanshinone IIa sulfonate pretreatment on high glucose-induced expression of fractalkine and apoptosis in human umbilical vein endothelial cells. Int. J. Clin. Exp. Med. 8 (4), 5279–5286. 26131102 PMC4483885

[B58] Li G. Lin X. R. Liu X. Lu C. Y. (2014). Advances in pharmacokinetics of Tan ⅡA and cryptotanshinone. J. Guangdong Med. Coll. 02, 236–238. 10.3969/j.issn.1005-4057.2014.02.047

[B59] Li W. Gao Z. Guan Q. L. (2023). Tan IIA mitigates vascular smooth muscle cell proliferation and migration induced by ox-LDL through the miR-137/TRPC3 axis. Kaohsiung J. Med. Sci. 39 (6), 596–604. 10.1002/kjm2.12663 36912285 PMC11895880

[B60] Li X. Du J. R. Yu Y. Bai B. Zheng X. Y. (2010). Tanshinone IIA inhibits smooth muscle proliferation and intimal hyperplasia in the rat carotid balloon-injured model through inhibition of MAPK signaling pathway. J. Ethnopharmacol. 129 (2), 273–279. 10.1016/j.jep.2010.03.021 20363310

[B61] Li Y. Chen F. Guo R. Jia S. Li W. Zhang B. (2020). Tanshinone ⅡA inhibits homocysteine-induced proliferation of vascular smooth muscle cells via miR-145/CD40 signaling. Biochem. Biophys. Res. Commun. 522 (1), 157–163. 10.1016/j.bbrc.2019.11.055 31757424

[B62] Li Y. Guo Y. Chen Y. Wang Y. You Y. Yang Q. (2015c). Establishment of an interleukin-1β-induced inflammation-activated endothelial cell-smooth muscle cell-mononuclear cell co-culture model and evaluation of the anti-inflammatory effects of tanshinone IIA on atherosclerosis. Mol. Med. Rep. 12 (2), 1665–1676. 10.3892/mmr.2015.3668 25936371 PMC4464412

[B63] Li Y. Huang J. Jiang Z. Zhong Y. Xia M. Wang H. (2016). MicroRNA-145 regulates platelet-derived growth factor-induced human aortic vascular smooth muscle cell proliferation and migration by targeting CD40. Am. J. Transl. Res. 8 (4), 1813–1825. 27186305 PMC4859910

[B64] Li Y. H. Xu Q. Xu W. H. Guo X. H. Zhang S. Chen Y. D. (2015a). Mechanisms of protection against diabetes-induced impairment of endothelium-dependent vasorelaxation by Tanshinone IIA. Biochim. Biophys. Acta 1850 (4), 813–823. 10.1016/j.bbagen.2015.01.007 25613563

[B65] Libby P. (2002). Inflammation in atherosclerosis. Nature 420 (6917), 868–874. 10.1038/nature01323 12490960

[B66] Libby P. (2021). The changing landscape of atherosclerosis. Nature 592 (7855), 524–533. 10.1038/s41586-021-03392-8 33883728

[B67] Lin R. Wang W. R. Liu J. T. Yang G. D. (2006). Protective effect of tanshinone IIA on human umbilical vein endothelial cell injured by hydrogen peroxide and its mechanism. J. Ethnopharmacol. 108 (2), 217–222. 10.1016/j.jep.2006.05.004 16797899

[B68] Liu W. Jiang J. Yan D. Li D. Li W. Ma Y. (2014b). Pentraxin 3 promotes oxLDL uptake and inhibits cholesterol efflux from macrophage-derived foam cells. Exp. Mol. Pathol. 96 (3), 292–299. 10.1016/j.yexmp.2014.03.007 24675235

[B69] Liu X. Guo C. Y. Ma X. J. Wu C. F. Zhang Y. Sun M. Y. (2015). Anti-inflammatory effects of tanshinone IIA on atherosclerostic vessels of ovariectomized ApoE mice are mediated by estrogen receptor activation and through the ERK signaling pathway. Cell Physiol. Biochem. 35 (5), 1744–1755. 10.1159/000373986 25832326

[B70] Liu Z. Wang J. Huang E. Gao S. Li H. Lu J. (2014a). Tanshinone IIA suppresses cholesterol accumulation in human macrophages: role of heme oxygenase-1. J. Lipid Res. 55 (2), 201–213. 10.1194/jlr.M040394 24302760 PMC3886659

[B71] Longo G. M. Xiong W. Greiner T. C. Zhao Y. Fiotti N. Baxter B. T. (2002). Matrix metalloproteinases 2 and 9 work in concert to produce aortic aneurysms. J. Clin. Invest. 110 (5), 625–632. 10.1172/JCI15334 12208863 PMC151106

[B72] Lou G. Hu W. Wu Z. Xu H. Yao H. Wang Y. (2020). Tanshinone II A attenuates vascular remodeling through klf4 mediated smooth muscle cell phenotypic switching. Sci. Rep. 10 (1), 13858. 10.1038/s41598-020-70887-1 32807822 PMC7431534

[B73] Lu J. Shan J. Liu N. Ding Y. Wang P. (2019). Tanshinone IIA can inhibit angiotensin II-induced proliferation and autophagy of vascular smooth muscle cells via regulating the MAPK signaling pathway. Biol. Pharm. Bull. 42 (11), 1783–1788. 10.1248/bpb.b19-00053 31391347

[B74] Lu M. Luo Y. Hu P. Dou L. Huang S. (2018). Tanshinone IIA inhibits AGEs-induced proliferation and migration of cultured vascular smooth muscle cells by suppressing ERK1/2 MAPK signaling. Iran. J. Basic Med. Sci. 21 (1), 83–88. 10.22038/IJBMS.2017.20100.5276 29372041 PMC5776441

[B75] Lutgens E. de Muinck E. D. Kitslaar P. J. Tordoir J. H. Wellens H. J. Daemen M. J. (1999). Biphasic pattern of cell turnover characterizes the progression from fatty streaks to ruptured human atherosclerotic plaques. Cardiovasc Res. 41 (2), 473–479. 10.1016/s0008-6363(98)00311-3 10341847

[B76] Lv B. Chen S. Tang C. Jin H. Du J. Huang Y. (2020). Hydrogen sulfide and vascular regulation - an update. J. Adv. Res. 27, 85–97. 10.1016/j.jare.2020.05.007 33318869 PMC7728588

[B77] Ma J. H. Zhao Q. Y. Wang Z. F. Li Z. Zhao L. (2015). Basic study on anti-atherosclerosis effect of tanshinone ⅡA. China Inf. J. Traditional Chin. Med. 06, 131–133. 10.3969/j.issn.1005-5304.2015.06.042

[B78] Ma T. Tian X. Zhang B. Li M. Wang Y. Yang C. (2022). Low-dose metformin targets the lysosomal AMPK pathway through PEN2. Nature 603 (7899), 159–165. 10.1038/s41586-022-04431-8 35197629 PMC8891018

[B79] Ma X. Zhang L. Gao F. Jia W. Li C. (2023). Salvia miltiorrhiza and Tanshinone IIA reduce endothelial inflammation and atherosclerotic plaque formation through inhibiting COX-2. Biomed. Pharmacother. 167, 115501. 10.1016/j.biopha.2023.115501 37713995

[B80] Major A. S. Harrison D. G. (2011). What fans the fire: insights into mechanisms of inflammation in atherosclerosis and diabetes mellitus. Circulation 124 (25), 2809–2811. 10.1161/CIRCULATIONAHA.111.070565 22184043 PMC3963008

[B81] Meng Z. Si C. Y. Teng S. Yu X. H. Li H. Y. (2019). Tanshinone IIA inhibits lipopolysaccharide-induced inflammatory responses through the TLR4/TAK1/NF-κB signaling pathway in vascular smooth muscle cells. Int. J. Mol. Med. 43 (4), 1847–1858. 10.3892/ijmm.2019.4100 30816448

[B82] Minguet S. Dopfer E. P. Pollmer C. Freudenberg M. A. Galanos C. Reth M. (2008). Enhanced B-cell activation mediated by TLR4 and BCR crosstalk. Eur. J. Immunol. 38 (9), 2475–2487. 10.1002/eji.200738094 18819072

[B83] Misra P. (2008). AMP activated protein kinase: a next generation target for total metabolic control. Expert Opin. Ther. Targets 12 (1), 91–100. 10.1517/14728222.12.1.91 18076373

[B84] Nagy L. Tontonoz P. Alvarez J. G. Chen H. Evans R. M. (1998). Oxidized LDL regulates macrophage gene expression through ligand activation of PPARgamma. Cell 93 (2), 229–240. 10.1016/s0092-8674(00)81574-3 9568715

[B85] Newby A. C. Zaltsman A. B. (2000). Molecular mechanisms in intimal hyperplasia. J. Pathol. 190 (3), 300–309. 10.1002/(SICI)1096-9896(200002)190:3<300::AID-PATH596>3.0.CO;2-I 10685064

[B86] Nizamutdinova I. T. Kim Y. M. Jin H. Son K. H. Lee J. H. Chang K. C. (2012). Tanshinone IIA inhibits TNF-α-mediated induction of VCAM-1 but not ICAM-1 through the regulation of GATA-6 and IRF-1. Int. Immunopharmacol. 14 (4), 650–657. 10.1016/j.intimp.2012.09.017 23085565

[B87] Noguchi K. Hamadate N. Matsuzaki T. Sakanashi M. Nakasone J. Uchida T. (2011). Increasing dihydrobiopterin causes dysfunction of endothelial nitric oxide synthase in rats *in vivo* . Am. J. Physiol. Heart Circ. Physiol. 301 (3), H721–H729. 10.1152/ajpheart.01089.2010 21622822

[B88] Ogawa S. Nakayama K. Nakayama M. Mori T. Matsushima M. Okamura M. (2010). Methylglyoxal is a predictor in type 2 diabetic patients of intima-media thickening and elevation of blood pressure. Hypertension 56 (3), 471–476. 10.1161/HYPERTENSIONAHA.110.156786 20644005

[B89] Ouimet M. Franklin V. Mak E. Liao X. Tabas I. Marcel Y. L. (2011). Autophagy regulates cholesterol efflux from macrophage foam cells via lysosomal acid lipase. Cell Metab. 13 (6), 655–667. 10.1016/j.cmet.2011.03.023 21641547 PMC3257518

[B90] Pasterkamp G. Schoneveld A. H. Hijnen D. J. de Kleijn D. P. Teepen H. van der Wal A. C. (2000). Atherosclerotic arterial remodeling and the localization of macrophages and matrix metalloproteases 1, 2 and 9 in the human coronary artery. Atherosclerosis 150 (2), 245–253. 10.1016/s0021-9150(99)00371-8 10856516

[B91] Qian S. Wang S. Fan P. Huo D. Dai L. Qian Q. (2012). Effect of Salvia miltiorrhiza hydrophilic extract on the endothelial biomarkers in diabetic patients with chronic artery disease. Phytother. Res. 26 (10), 1575–1578. 10.1002/ptr.4611 22318996

[B92] Qian Y. He Y. Qiong A. Zhang W. (2023). Tanshinone IIA regulates MAPK/mTOR signal-mediated autophagy to alleviate atherosclerosis through the miR-214-3p/atg16l1 Axis. Int. Heart J. 64 (5), 945–954. 10.1536/ihj.23-087 37778998

[B93] Qin Y. Zheng B. Yang G. S. Zhou J. Yang H. J. Nie Z. Y. (2020). Tanshinone ⅡA inhibits VSMC inflammation and proliferation *in vivo* and *in vitro* by downregulating miR-712-5p expression. Eur. J. Pharmacol. 880, 173140. 10.1016/j.ejphar.2020.173140 32387370

[B94] Ross R. Glomset J. A. (1976). The pathogenesis of atherosclerosis (first of two parts). N. Engl. J. Med. 295 (7), 369–377. 10.1056/NEJM197608122950707 819830

[B95] Shang T. Liu Z. Zhou M. Zarins C. K. Xu C. Liu C. j. (2012). Inhibition of experimental abdominal aortic aneurysm in a rat model by way of tanshinone IIA. J. Surg. Res. 178 (2), 1029–1037. 10.1016/j.jss.2012.04.068 22640888

[B96] Shi C. Zhu X. Wang J. Long D. (2014). Tanshinone IIA promotes non-amyloidogenic processing of amyloid precursor protein in platelets via estrogen receptor signaling to phosphatidylinositol 3-kinase/Akt. Biomed. Rep. 2 (4), 500–504. 10.3892/br.2014.263 24944795 PMC4051464

[B97] Shi X. P. Li J. Pan W. Lv Q. Z. Safety analysis of tanshinone IIA sodium sulfonate injection used in 125 inpatients of our hospital [J]. Shanghai Pharm.,2016,37(19):58–63.

[B98] Shi Y. Jiang T. Y. Wang S. L. (2007). Development of tan IIA and its preparations. World Clin. Med. 07, 439–441+443.

[B99] Shu D. Chen X. J. Zhang L. (2015). Literature analysis of adverse reactions of sodium Tan II A sulfonate injection. Chin. J. Pract. Med. 9, 178–179. 10.14163/j.cnki.11-5547/r.2015.09.127

[B100] Stangl V. Lorenz M. Meiners S. Ludwig A. Bartsch C. Moobed M. (2004). Long-term up-regulation of eNOS and improvement of endothelial function by inhibition of the ubiquitin-proteasome pathway. FASEB J. 18 (2), 272–279. 10.1096/fj.03-0054com 14769821

[B101] Stocker R. Perrella M. A. (2006). Heme oxygenase-1: a novel drug target for atherosclerotic diseases? Circulation 114 (20), 2178–2189. 10.1161/CIRCULATIONAHA.105.598698 17101869

[B102] Stolla M. Pelisek J. von Brühl M. L. Schäfer A. Barocke V. Heider P. (2012). Fractalkine is expressed in early and advanced atherosclerotic lesions and supports monocyte recruitment via CX3CR1. PLoS One 7 (8), e43572. 10.1371/journal.pone.0043572 22916279 PMC3423360

[B103] Tan Y. L. Ou H. X. Zhang M. Gong D. Zhao Z. W. Chen L. Y. (2019). Tanshinone IIA promotes macrophage cholesterol efflux and attenuates atherosclerosis of apoE-/- mice by omentin-1/ABCA1 pathway. Curr. Pharm. Biotechnol. 20 (5), 422–432. 10.2174/1389201020666190404125213 30947667

[B104] Tang C. Wu A. H. Xue H. L. Wang Y. j. (2007). Tanshinone IIA inhibits endothelin-1 production in TNF-alpha-induced brain microvascular endothelial cells through suppression of endothelin-converting enzyme-1 synthesis. Acta Pharmacol. Sin. 28 (8), 1116–1122. 10.1111/j.1745-7254.2007.00598.x 17640471

[B105] Tang C. Xue H. L. Bai C. L. Fu R. (2011b). Regulation of adhesion molecules expression in TNF-α-stimulated brain microvascular endothelial cells by tanshinone IIA: involvement of NF-κB and ROS generation. Phytother. Res. 25 (3), 376–380. 10.1002/ptr.3278 20687137

[B106] Tang F. T. Cao Y. Wang T. Q. Wang L. J. Guo J. Zhou X. S. (2011a). Tanshinone IIA attenuates atherosclerosis in ApoE(-/-) mice through down-regulation of scavenger receptor expression. Eur. J. Pharmacol. 650 (1), 275–284. 10.1016/j.ejphar.2010.07.038 20854809

[B107] Thum T. Fraccarollo D. Schultheiss M. Froese S. Galuppo P. Widder J. D. (2007). Endothelial nitric oxide synthase uncoupling impairs endothelial progenitor cell mobilization and function in diabetes. Diabetes 56 (3), 666–674. 10.2337/db06-0699 17327434

[B108] Vlassara H. Bucala R. Striker L. (1994). Pathogenic effects of advanced glycosylation: biochemical, biologic, and clinical implications for diabetes and aging. Lab. Invest. 70 (2), 138–151. 8139257

[B109] Wang B. Ge Z. Cheng Z. Zhao Z. (2017). Tanshinone IIA suppresses the progression of atherosclerosis by inhibiting the apoptosis of vascular smooth muscle cells and the proliferation and migration of macrophages induced by ox-LDL. Biol. Open 6 (4), 489–495. 10.1242/bio.024133 28412716 PMC5399561

[B110] Wang H. R. Li D. Weng H. C. Yue X. (2014). Epidemiological study on adverse reactions of sodium tanshinone ⅡA sulfonate injection. China Pharm. Ind. 14, 43–45.

[B111] Wang J. He X. Chen W. Zhang N. Guo J. Liu J. (2020b). Tanshinone IIA protects mice against atherosclerotic injury by activating the TGF-β/PI3K/Akt/eNOS pathway. Coron. Artery Dis. 31 (4), 385–392. 10.1097/MCA.0000000000000835 31842027 PMC7192539

[B112] Wang J. Liu W. Lu W. Luo X. Lin Y. Liu S. (2022). Sodium tanshinone IIA sulfonate enhances the BMP9-BMPR2-Smad1/5/9 signaling pathway in rat pulmonary microvascular endothelial cells and human embryonic stem cell-derived endothelial cells. Biochem. Pharmacol. 199, 114986. 10.1016/j.bcp.2022.114986 35276216

[B113] Wang J. Zhang Y. Feng X. Du M. Li S. Chang X. (2023). Tanshinone IIA alleviates atherosclerosis in LDLR-/- mice by regulating efferocytosis of macrophages. Front. Pharmacol. 14, 1233709. 10.3389/fphar.2023.1233709 37886125 PMC10598641

[B114] Wang M. Li W. Chang G. Q. Ye C. S. Ou J. S. Li X. X. (2011). MicroRNA-21 regulates vascular smooth muscle cell function via targeting tropomyosin 1 in arteriosclerosis obliterans of lower extremities. Arterioscler. Thromb. Vasc. Biol. 31 (9), 2044–2053. 10.1161/ATVBAHA.111.229559 21817107

[B115] Wang N. Zhang X. Ma Z. Niu J. Ma S. Wenjie W. (2020a). Combination of tanshinone IIA and astragaloside IV attenuate atherosclerotic plaque vulnerability in ApoE(-/-) mice by activating PI3K/AKT signaling and suppressing TRL4/NF-κB signaling. Biomed. Pharmacother. 123, 109729. 10.1016/j.biopha.2019.109729 31887543

[B116] Wang X. X. Yang J. X. Pan Y. Y. Zhang Y. F. (2015). Protective effects of tanshinone ⅡA on endothelial progenitor cells injured by tumor necrosis factor-α. Mol. Med. Rep. 12 (3), 4055–4062. 10.3892/mmr.2015.3969 26095681 PMC4526031

[B117] Watanabe K. Watanabe R. Konii H. Shirai R. Sato K. Matsuyama T. A. (2016). Counteractive effects of omentin-1 against atherogenesis. Cardiovasc Res. 110 (1), 118–128. 10.1093/cvr/cvw016 26790473

[B118] Wen J. Chang Y. Huo S. Li W. Huang H. Gao Y. (2020). Tanshinone IIA attenuates atherosclerosis via inhibiting NLRP3 inflammasome activation. Aging (Albany NY) 13 (1), 910–932. 10.18632/aging.202202 33290264 PMC7835056

[B119] Wu W. Y. Yan H. Wang X. B. Gui Y. z. Gao F. Tang X. l. (2014). Sodium tanshinone IIA silate inhibits high glucose-induced vascular smooth muscle cell proliferation and migration through activation of AMP-activated protein kinase. PLoS One 9 (4), e94957. 10.1371/journal.pone.0094957 24739942 PMC3989257

[B120] Xu S. Little P. J. Lan T. Huang Y. Le K. Wu X. (2011). Tanshinone II-A attenuates and stabilizes atherosclerotic plaques in apolipoprotein-E knockout mice fed a high cholesterol diet. Arch. Biochem. Biophys. 515 (1-2), 72–79. 10.1016/j.abb.2011.08.006 21889487

[B121] Xuan Y. Gao Y. Huang H. Wang X. Cai Y. Luan Q. X. (2017). Tanshinone IIA attenuates atherosclerosis in apolipoprotein E knockout mice infected with porphyromonas gingivalis. Inflammation 40 (5), 1631–1642. 10.1007/s10753-017-0603-8 28646427

[B122] Xuan Y. Yu C. Ni K. Congcong L. Lixin Q. Qingxian L. (2023). Protective effects of tanshinone IIA on Porphyromonas gingivalis-induced atherosclerosis via the downregulation of the NOX2/NOX4-ROS mediation of NF-κB signaling pathway. Microbes Infect., 105177. 10.1016/j.micinf.2023.105177 37392987

[B123] Yakubu M. A. Leffler C. W. (2002). L-type voltage-dependent Ca2+ channels in cerebral microvascular endothelial cells and ET-1 biosynthesis. Am. J. Physiol. Cell Physiol. 283 (6), C1687–C1695. 10.1152/ajpcell.00071.2002 12388093 PMC2924154

[B124] Yan Q. Mao Z. Hong J. Gao K. Niimi M. Mitsui T. (2021). Tanshinone IIA stimulates cystathionine γ-lyase expression and protects endothelial cells from oxidative injury. Antioxidants (Basel) 10 (7), 1007. 10.3390/antiox10071007 34201701 PMC8300834

[B125] Yang C. Lei X. Li J. (2019). Tanshinone IIA reduces oxidized low-density lipoprotein-induced inflammatory responses by downregulating microRNA-33 in THP-1 macrophages. Int. J. Clin. Exp. Pathol. 12 (10), 3791–3798. 31933767 PMC6949767

[B126] Yang J. X. Pan Y. Y. Ge J. H. Chen B. Mao W. Qiu Y. G. (2016). Tanshinone II A attenuates TNF-α-Induced expression of VCAM-1 and ICAM-1 in endothelial progenitor cells by blocking activation of NF-κB. Cell Physiol. Biochem. 40 (1-2), 195–206. 10.1159/000452537 27855363

[B127] Yang J. X. Pan Y. Y. Zhao Y. Y. Wang X. X. (2013). Endothelial progenitor cell-based therapy for pulmonary arterial hypertension. Cell Transpl. 22 (8), 1325–1336. 10.3727/096368912X659899 23295102

[B128] Yu Z. L. Wang J. N. Wu X. H. Xie H. J. Han Y. Guan Y. T. (2015). Tanshinone IIA prevents rat basilar artery smooth muscle cells proliferation by inactivation of PDK1 during the development of hypertension. J. Cardiovasc Pharmacol. Ther. 20 (6), 563–571. 10.1177/1074248415574743 25736282

[B129] Yuan L. Li Q. Zhang Z. Liu Q. Wang X. Fan L. (2020). Tanshinone IIA inhibits the adipogenesis and inflammatory response in ox-LDL-challenged human monocyte-derived macrophages via regulating miR-130b/WNT5A. J. Cell Biochem. 121 (2), 1400–1408. 10.1002/jcb.29375 31512787

[B130] Zhang E. Wu Y. (2013). MicroRNAs: important modulators of oxLDL-mediated signaling in atherosclerosis. J. Atheroscler. Thromb. 20 (3), 215–227. 10.5551/jat.15180 23064493

[B131] Zhang Y. Mao H. P. Fan G. W. (2019). Research progress on the pharmacological effects of tanshinone IIA. J. Tianjin Univ. Traditional Chin. Med. 01, 15–19. 10.11656/j.issn.1673-9043.2019.01.05

[B132] Zhao A. J. Liu X. L. Fan X. X. (2023). Pharmacokinetics and pharmacodynamics of a single intramuscular injection of sodium Tan IIA sulfonate in healthy subjects. Wisdom Health 21 (24), 148–150. 10.19335/j.cnki.2096-1219.2021.24.050

[B133] Zhao D. Tong L. Zhang L. Li H. Wan Y. Zhang T. (2016). Tanshinone II A stabilizes vulnerable plaques by suppressing RAGE signaling and NF-κB activation in apolipoprotein-E-deficient mice. Mol. Med. Rep. 14 (6), 4983–4990. 10.3892/mmr.2016.5916 27840935 PMC5355755

[B134] Zhou W. J. Gui Q. F. Wu Y. Yang Y. M. (2015). Tanshinone IIA protects against methylglyoxal-induced injury in human brain microvascular endothelial cells. Int. J. Clin. Exp. Med. 8 (2), 1985–1992. 25932127 PMC4402774

[B135] Zhou Z. W. Xie X. L. Zhou S. F. Li C. G. (2012). Mechanism of reversal of high glucose-induced endothelial nitric oxide synthase uncoupling by tanshinone IIA in human endothelial cell line EA.hy926. Eur. J. Pharmacol. 697 (1-3), 97–105. 10.1016/j.ejphar.2012.09.051 23063542

[B136] Zhou Z. Y. Shi W. T. Zhang J. Zhao W. R. Xiao Y. Zhang K. Y. (2023). Sodium tanshinone IIA sulfonate protects against hyperhomocysteine-induced vascular endothelial injury via activation of NNMT/SIRT1-mediated NRF2/HO-1 and AKT/MAPKs signaling in human umbilical vascular endothelial cells. Biomed. Pharmacother. 158, 114137. 10.1016/j.biopha.2022.114137 36525817

[B137] Zhu H. Chen Z. Ma Z. Tan H. Xiao C. Tang X. (2017a). Tanshinone IIA protects endothelial cells from H₂O₂-Induced injuries via PXR activation. Biomol. Ther. Seoul. 25 (6), 599–608. 10.4062/biomolther.2016.179 28173640 PMC5685429

[B138] Zhu J. Chen H. Guo J. Zha C. Lu D. (2022). Sodium tanshinone IIA sulfonate inhibits vascular endothelial cell pyroptosis via the AMPK signaling pathway in atherosclerosis. J. Inflamm. Res. 15, 6293–6306. 10.2147/JIR.S386470 36408328 PMC9673812

[B139] Zhu J. Xu Y. Ren G. Hu X. Wang C. Yang Z. (2017b). Tanshinone IIA Sodium sulfonate regulates antioxidant system, inflammation, and endothelial dysfunction in atherosclerosis by downregulation of CLIC1. Eur. J. Pharmacol. 815, 427–436. 10.1016/j.ejphar.2017.09.047 28970012

[B140] Zhu L. Sun G. Zhang H. Zhang Y. Chen X. Jiang X. (2009). PGC-1alpha is a key regulator of glucose-induced proliferation and migration in vascular smooth muscle cells. PLoS One 4 (1), e4182. 10.1371/journal.pone.0004182 19142226 PMC2615131

[B141] Zhu Y. Xian X. Wang Z. Bi Y. Chen Q. Han X. (2018). Research progress on the relationship between atherosclerosis and inflammation. Biomolecules 8 (3), 80. 10.3390/biom8030080 30142970 PMC6163673

